# An allosteric photoredox catalyst inspired by photosynthetic machinery

**DOI:** 10.1038/ncomms7541

**Published:** 2015-03-30

**Authors:** Alejo M. Lifschitz, Ryan M. Young, Jose Mendez-Arroyo, Charlotte L. Stern, C. Michael McGuirk, Michael R. Wasielewski, Chad A. Mirkin

**Affiliations:** 1 Department of Chemistry and International Institute for Nanotechnology, Northwestern University, 2145 Sheridan Road, Evanston, Illinois 60208, USA; 2 Argonne-Northwestern Solar Energy Research (ANSER) Center, Northwestern University, Evanston, Illinois 60208, USA

## Abstract

Biological photosynthetic machinery allosterically regulate light harvesting via conformational and electronic changes at the antenna protein complexes as a response to specific chemical inputs. Fundamental limitations in current approaches to regulating inorganic light-harvesting mimics prevent their use in catalysis. Here we show that a light-harvesting antenna/reaction centre mimic can be regulated by utilizing a coordination framework incorporating antenna hemilabile ligands and assembled via a high-yielding, modular approach. As in nature, allosteric regulation is afforded by coupling the conformational changes to the disruptions in the electrochemical landscape of the framework upon recognition of specific coordinating analytes. The hemilabile ligands enable switching using remarkably mild and redox-inactive inputs, allowing one to regulate the photoredox catalytic activity of the photosynthetic mimic reversibly and *in situ*. Thus, we demonstrate that bioinspired regulatory mechanisms can be applied to inorganic light-harvesting arrays displaying switchable catalytic properties and with potential uses in solar energy conversion and photonic devices.

## Abstract (web summary)

Photosynthetic systems regulate light harvesting via structural and electronic control of antenna proteins. Here, the authors report a light-harvesting antenna/reaction centre mimic that can be allosterically regulated using mild and redox-inactive inputs, via a coordination framework with hemilabile ligands.

## Graphical abstract

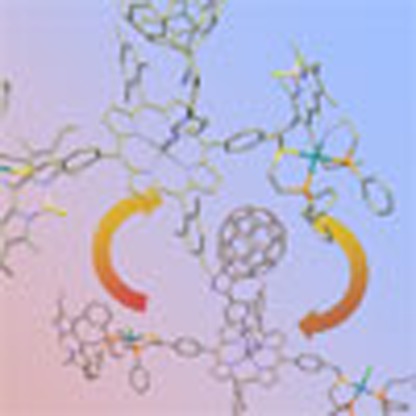

Allosteric and recognition-based control of light harvesting in natural photosynthetic systems is critical to sustaining a balanced chemical and redox environment inside cells^1^,^2^,^3^,^4^,^5^. Achieving this kind of regulation with artificial light-harvesting systems would allow one to control the production of redox and energetic feedstock from light and, subsequently, to apply chemical regulation of photosynthetic activity to systems that extend beyond the reach of biology^6^. Indeed, a general approach for the chemical regulation of inorganic mimics would expand their application in solar energy conversion and photonic devices, as well as enabling their use in signal amplification, catalytic switches and stimuli-responsive materials^7^,^8^,^9^,^10^,^11^,^12^,^13^,^14^,^15^,^16^. Furthermore, achieving enzyme-like regulatory properties will become useful as artificial constructs begin to be studied in the context of light-harvesting hybrid systems comprising inorganic and biological units^17^. While there is a rich variety of approaches for controlling light harvesting in natural systems, triggering conformational changes in antenna protein complexes as a response to specific chemical inputs (such as pH fluctuations) represents a common strategy that can form the basis for regulation in inorganic systems^18^,^19^,^20^. Specifically, allosterically triggered conformational changes in antenna protein complexes induce kinetically fast quenching mechanisms (for example, charge transfer and electron transfer) by changing the electronic structure of the embedded dyes or by affecting their electronic coupling with other photoactive moieties^21^,^22^,^23^.

The development of a general route for switchable inorganic mimics that can be applied to catalysis and device applications will require a novel approach which, similar to regulatory strategies in nature, allows for inducing large changes in light-harvesting efficiency using mild and selective inputs. Molecular arrays previously studied within this context have enabled remarkable self-regulation and switching of light-harvesting efficiency, yet they cannot be exploited for catalyst control given their intrinsic design limitations. For instance, the activity of light-harvesting antenna/reaction centre mimics has been regulated by appending photochromic switches that can photoisomerize into energy sinks^24^,^25^,^26^,^27^. Since the switching inputs cannot address the light-harvesting array and the switching unit independently and photoisomerization quantum yields are low, this approach is best suited to cap the overall yield of light harvesting at high light intensities rather than to create ON/OFF switches. Chemical switching via pH fluctuations has also been explored as a means to regulate light harvesting. Protonation can be used to tune the electronics of the light-harvesting units or to trigger the formation of energy sinks^28^,^29^. However, the need for large changes in pH results in switching inputs that are far more likely to affect the nature of the redox reactions catalysed by the light-harvesting array than to chemically switch the array itself, thus hampering the application of this approach in catalysis. Furthermore, the regulatory strategies mentioned above rely on quenching via the introduction of a divergent energy transfer step, which does not exhibit intrinsically faster kinetics than the light-harvesting photophysical pathways they set out to out-compete, often limiting switching ratios. Regulation of supramolecular structure in the reaction centre mimics represents a promising alternative since supramolecular switches can be operated using mild coordination inputs^30^ or competitive inhibitors^31^. While coordination chemistry has been widely used to regulate molecular logic gates, chemosensors and to manage light-driven charge separation, there are no examples in which allosteric coordination inputs are used to shuttle a photoredox catalyst between active and inactive states reversibly as observed in biological photosynthetic systems.

Against this backdrop, we describe a set of allosterically controlled light-harvesting switches that incorporate photoactive components reminiscent of photosynthetic machinery and that can be used to reversibly regulate photoredox catalytic activity *in situ* and reversibly ((**1–3**)**-ImC**_**60**,_ Fig. 1). In particular, we employ an established model for a light-harvesting antenna/reaction centre mimic composed of Bodipy, porphyrin and fullerene units, which are hereby embedded in a Rh(I) coordination complex whose interaction with allosteric coordinating effectors can be used to disrupt the framework’s electrochemistry. This regulatory approach is based on reversibly controlling antenna hemilabile ligand coordination to an allosteric Rh(I) receptor, enabling a rare example of chemical switching via coupled electronic and structural changes^12^,^32^,^33^,^34^ typical of photosynthetic systems and proteins^35^,^36^,^37^. The use of a hemilabile coordination chemistry approach enables one to interconvert between active and inactive species in bulk and quantitatively using mild and redox-inactive chemical inputs that render the system compatible with a large array of redox processes. These structures are rapidly and reliably assembled via the weak-link approach (WLA)^38^, a synthetic strategy that exploits orthogonal coordination interactions between structural metal centres and functional hemilabile ligands. Analogous to regulation strategies in photosystem II, the switching of catalytic activity depends on regulating energy transfer from the antennae to a reaction centre mimic exploiting an intrinsically faster quenching mechanism. In particular, switch (**1–3**)**-ImC**_**60**_ relies on utilizing coordinating effectors that control the redox potential of the antennae relative to the structural metal centres. Thus, photoinduced electron transfer (PeT)^39^, which here arises in the sub-picosecond range^40^, is used to out-compete picosecond-range energy transfer from the excited antenna to the catalytically active reaction centre. While the quenched state also results in light-driven charge separation, the redox potential of the species it is comprised of does not allow for catalytic turnover. Consequently, we are able to achieve light-harvesting switching ratios of up to 39-fold in a bulk sample. Importantly, the activation of photoredox activity occurs selectively upon binding of allosteric effectors with the proper electron-donating ability. Thus, we demonstrate that coordination chemistry can be exploited to surmount structural, regulatory and selectivity challenges relevant to the design of light-harvesting switches, enabling their potential use in catalytic, photonic and energy conversion application devices^13^,^14^,^15^,^41^,^42^,^43^.

## Results

### Design and synthesis of inactive coordination framework

**Figure 2:**
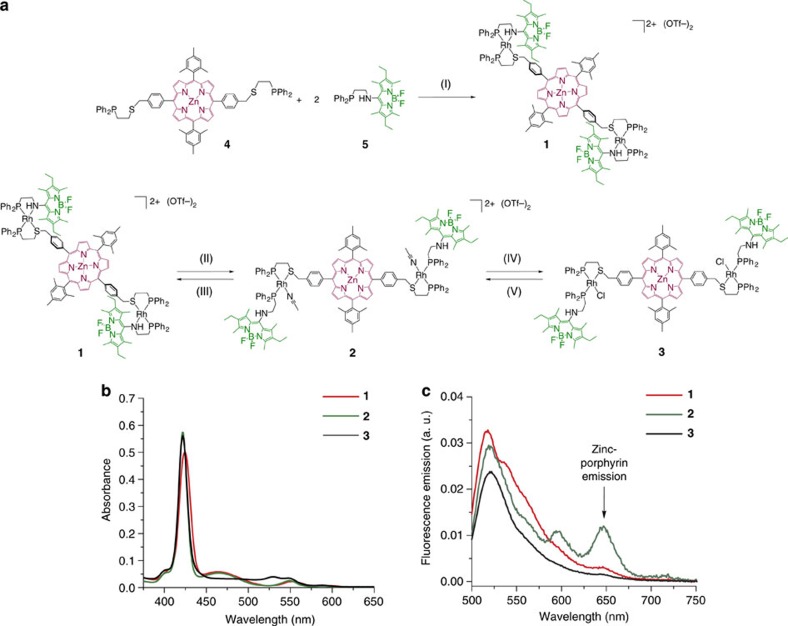
Allosteric energy transfer switch operated via coordination chemistry. (**a**) Synthesis of WLA switch and toggling between coordination states with acetonitrile and chloride. Reaction conditions: (I) Rh_2_Cl_2_(COE), Tl(OTf), CH_2_Cl_2_; (II) 50 μl CH_3_CN, CH_2_Cl_2_; (III) evaporation and redissolution in CH_2_Cl_2_; (IV) 2 equiv. N(n-Bu)_4_Cl, CH_2_Cl_2_; (V) 2 equiv. Tl(OTf), CH_2_Cl_2_. (**b**) Absorbance spectra and (**c**) fluorescence emission spectra (λ_ex_=480 nm).

In the past decade, many molecular photoactive ensembles that mimic the interactions between light-harvesting antenna and the reaction centre of photosynthetic systems^44^ have been built. This is typically achieved by the incorporating an antenna chromophore whose ground state can be selectively excited at a given wavelength, which triggers excitation energy transfer to an electron transfer pair wherein that energy drives the formation of a charge-separated state. In these constructs, Bodipy has often been employed as the antenna since its HOMO-LUMO (highest occupied molecular orbital and lowest unoccupied molecular orbital) gap and redox properties can be readily tuned via synthetic modifications around its core. Furthermore, Bodipy can be covalently appended to porphyrin energy acceptors, often displaying high energy transfer efficiencies^45^. Incorporation of an electron acceptor into Bodipy–porphyrin ensembles can be achieved via a variety of covalent or supramolecular approaches^30^, yet coordination of a nitrogen base-functionalized fullerene to a metallated porphyrin has proven a synthetically simpler approach that allows for PeT from the excited porphyrin to occur with high efficiency^46^,^47^. Herein, we employed a coordination-driven strategy based on the WLA to assemble a structurally switchable Bodipy–porphyrin-fullerene framework that circumvents the need for complex synthetic procedures. Towards this end, we assembled zinc porphyrin and Bodipy moieties functionalized with hemilabile coordination ligands (**4** and **5**, Supplementary Figs 1 and 2) around Rh(I) structural centres in a one-pot, high-yielding procedure leading to **1** (Fig. 2a). This is possible due to the WLA and the halide-induced ligand rearrangement reaction^48^, which serve to selectively arrange the ligands around Rh(I) yielding a heteroligated complex with porphyrin and Bodipy ligands chelated in a *cis* conformation. Diagnostic ^31^P[^1^H] nuclear magnetic resonance (NMR) spectroscopy (*δ*: 66.8, *J*_P-P_=38 Hz, *J*_P-Rh_=178 Hz and 50.0, *J*_P-P_=38 Hz, *J*_P-Rh_=166 Hz), as well as the solid-state structure of a related compound in which the antennae are modified with a thioether spacer (**X1**, Supplementary Fig. 3), support this structural assignment. While the quality of the crystal structure is low (*R1*=0.14), the structure provides a good overall picture of the coordination mode of **1** with two bidentate hemilabile ligands coordinated to each Rh(I) centre in a *cis* fashion. Chelation of Bodipy ligand **5** to the charged metal centre in **1** serves to shift the antenna’s oxidation potential to higher values by 180 mV (Supplementary Figs 4–6 and Supplementary Table 1), enabling quenching of the excited Bodipy via PeT from Rh(I)^40^. This process can be observed to arise via transient absorption (TA) spectroscopy with a rate of *k*_PeT_=2.0 × 10^12^ s^−1^ in a model Bodipy-Rh(I) complex **S1**, whereas energy transfer in **1** takes place with a rate of *k*_EnT_=8.3 × 10^10^ s^−1^ (see Supplementary Figs 7–13). As a result, excitation of the antenna at 480 nm only results in minimal energy transfer to the porphyrin unit, as revealed by the fluorescence emission spectra of **1** (Fig. 2c).

### Effects of allosteric effector binding

By displacing the Bodipy hemilabile ligand with a neutral allosteric effector and expanding the complex, the oxidation potential of the antenna can be shifted to more negative potentials while maintaining the oxidation potential of Rh(I) (Supplementary Fig. 5). Thus, coordination of acetonitrile in **1** to give **2** (^31^P[^1^H] NMR *δ*: 69.2, *J*_P-P_=44 Hz, *J*_P-Rh_=172 Hz and 25.8, *J*_P-P_=44 Hz, *J*_P-Rh_=160 Hz) disables PeT from Rh(I) and an increase in energy transfer efficiency to the porphyrin ensues (Fig. 2c). The change in energy transfer efficiency can be approximated from changes in the lifetime of the excited antenna in **1** and **2**, determined via near-infrared TA (NIR-TA) spectroscopy (Supplementary Figs 14–19), relative to previously reported Rh(I) complexes in which the zinc porphyrin unit is replaced by a benzyl group (complexes **S1** and **S2**, see Supplementary Methods)^40^. For example, comparison of the antenna excited state lifetimes (*τ*) in **1** and its model complex **S1** allows us to approximate the energy transfer efficiency (*E*) using the equation *E*=1-(*τ*_**1**_/*τ*_**S1**_). When taking into account the change in absorption cross section following acetonitrile coordination (Fig. 2a), our data yield an 11-fold switching ratio (Supplementary Table 2). This value is particularly remarkable given the strong distance dependence of energy transfer^49^, suggesting that modulating PeT serves to enhance energy transfer even as the components are spatially further apart.

While energy transfer is enhanced via coordination of a neutral acetonitrile effector to **1**, the incorporation of anionic chloride effectors has the opposite effect. Indeed, the formation of **3** (^31^P[^1^H] NMR *δ*: 71.4, J_P-P_=40 Hz, J_P-Rh_=184 Hz and 30.8, J_P-P_=40 Hz, J_P-Rh_=166 Hz) via the addition of two equivalents of N(n-Bu)_4_Cl to either **1** or **2** results in minimal porphyrin-based emission upon excitation of the antenna (Fig. 2c). The electrochemical basis for the substrate specificity is derived from the fact that chloride lowers the Rh(I) oxidation potential (*ΔV*=−553 mV) more than it shifts Bodipy’s (Supplementary Fig. 6), thus enhancing rather than disabling the thermodynamic driving force for PeT from Rh(I) to Bodipy. By making the same approximations as above, we can estimate that the energy transfer efficiency in **3** is 8 times lower than in **1** and 39 times lower than in **2** (Supplementary Figs 20–25 and Supplementary Table 2). Importantly, the benzyl spacer in ligand **4** ensures that through-bond electronic communication between the porphyrin and the rest of the complex is reduced. Therefore, the lifetime of the excited energy acceptor is roughly constant following excitation of the antenna (1,274±207 ps in **1**, 1,126±67 in **2** and 1,121±90 ps in **3**, *λ*=1,280 nm) regardless of the changes in coordination mode. These values suggest that PeT from Rh(I) to Bodipy is indeed the primary contributor to the toggling of energy transfer.

### Antenna-reaction centre mimic assembly

Axial coordination to the porphyrin’s Zn(II) in **1**–**3** can be exploited to incorporate an imidazole-modified fullerene moiety (**ImC**_**60**_, ref. 50), thus giving (**1–3**)**-ImC**_**60**_. The Zn(II)-imidazole association constant is within the same order of magnitude for the three complexes (*K*_*a*_=1.8 × 10^5^±5% for **1**, *K*_*a*_=1.1 × 10^5^±18% for **2** and *K*_*a*_=2.7 × 10^5^±13% for **3**, see Supplementary Figs 26–28), owing to the fact that the porphyrin ligand exhibits a structural kink introduced by the benzyl bridge. This kink renders the axial coordination site physically accessible regardless of coordination changes at the Rh(I) centre (see solid-state structure of **4** and **X1**, Supplementary Figs 1 and 3). While coordination to Zn(II) requires an excess of **ImC**_**60**_, the excess does not interfere with coordination processes at the Rh(I) centre. Indeed, ^31^P[^1^H] NMR and optical spectra of (**1–3**)-**ImC**_**60**_ show that the coordination of hemilabile ligands **4** and **5** is not perturbed even after the addition of 40 equiv. of **ImC**_**60**_ and that the framework can be interconverted reversibly and *in situ* between the coordination states (Supplementary Figs 29–32). Thus, a series of orthogonal coordination behaviours particular to each ligand (Bodipy, porphyrin and fullerene) allow for the straightforward assembly of a switchable light-harvesting antenna, reaction centre mimic.

### Regulation of charge separation in the reaction centre mimic

**Figure 3:**
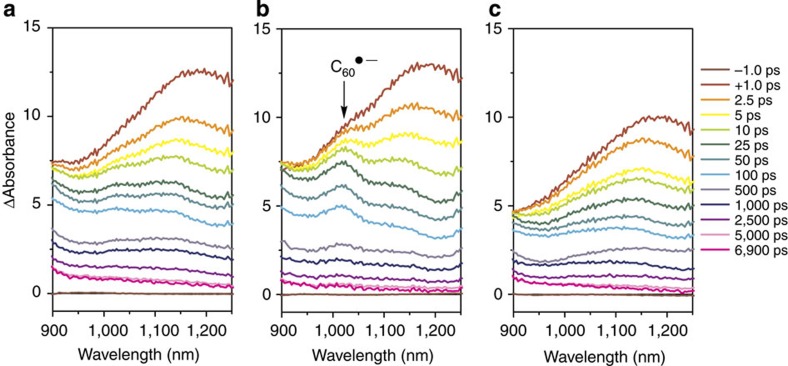
NIR-TA spectra of (**1–3**)-**ImC**_**60**_. Excitation of 3 μM solutions of **1** (**a**), **2** (**b**) and **3** (**c**) with 1.1-μJ laser pulses in the presence of 10 equiv. of **ImC**_**60**_ in CH_2_Cl_2_ (λ_ex_=477 nm).

The optical properties of (**1–3**)-**ImC**_**60**_ were studied to evaluate their ability to form catalytically active, charge-separated states. In related Bodipy–zinc porphyrin-fullerene molecular assemblies, it has been observed that the rate of electron transfer from the excited porphyrin to the fullerene is significantly faster than the rate of energy transfer from Bodipy to the porphyrin, which results in a large decrease in the porphyrin-based fluorescence quantum yield^45^,^46^. In the case of (**1–3**)-**ImC**_**60**,_ steady-state fluorescence emission spectra of the three supramolecular structures reveal that the porphyrin excited state is largely depleted following the incorporation of the fullerene moiety and excitation of the antenna (Supplementary Fig. 33). This suggests that, while the formation efficiency of the porphyrin excited state is different in the three coordination modes, the excited porphyrin, however much of it is produced, will undergo electron transfer to the fullerene with high efficiency in all cases. Further, when the antennae are excited in equimolar samples of (**1–3**)-**ImC**_**60**_ using the same excitation intensity, the absorbance resulting from the fullerene radical anion at 1,010 nm, detected via NIR-TA spectroscopy, is noticeably different (Fig. 3). In particular, absorption by the fullerene anion is markedly larger in the case of **2**-**ImC**_**60**_ (Fig. 3b) relative to both **1**-**ImC**_**60**_ and **3**-**ImC**_**60**_ (Fig. 3a,c). This suggests that the PeT switch above, which operates simply by introducing and abstracting allosteric effectors of the proper charge, can also regulate charge separation within the central reaction centre mimic.

### Allosteric control of photoredox catalytic activity

**Figure 4:**
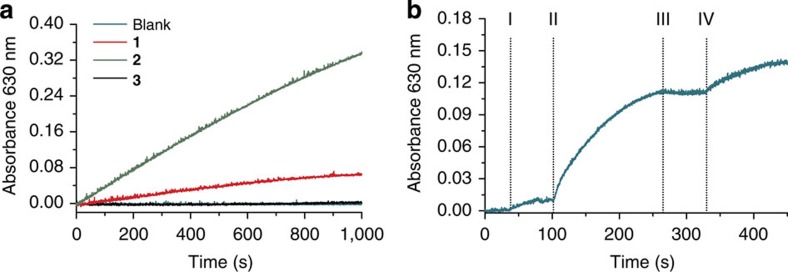
Catalytic reduction of methyl viologen in the presence of (**1–3**)**-ImC**_**60**_. (**a**) Changes in the absorbance at 630 nm in the presence of 1 μM **1**, **2**, **3** or no complex (blank) with 10 equiv. of **ImC**_**60**_ (λ_ex_=480 nm, 0.8 mW) in CH_2_Cl_2_. (**b**) Changes in the absorbance at 630 nm in the presence of 5 μM CH_2_Cl_2_ solution of **1** with 10 equiv. of **ImC**_**60**_ on: (I) turning excitation source on (λ_ex_=480 nm, 0.3 mW), (II) addition of acetonitrile (1 drop), (III) addition of chloride (2 equiv.), and (IV) addition of Tl(OTf; 6 equiv.).

To examine whether the modulation of charge-separation efficiency translates into the regulation of photoredox activity, the reduction of methyl viologen by 1-benzyl-1,4-dihydronicotinamide was investigated in the presence of (**1–3**)-**ImC**_**60**_ and an excitation source. This electron transfer process, which is energetically uphill in terms of the two reactants, stores about 1 eV of the energy absorbed by the light-harvesting Bodipy antenna^51^,^52^. Indeed, in the presence of the active species **2**-**ImC**_**60**_, catalytic turnover is observed (*k*=2.8 × 10^−7^±0.1% s^−1^), as evidenced by absorbance changes in the mixture at 630 nm (Fig. 4a and Supplementary Fig. 34), characteristic of the reduced methyl viologen species^53^. These bioinspired species are thus able to make use of dihydronicotinamide as a sacrificial reductant, which itself is the basis for the reduced nicotinamide adenine dinucleotide, a common redox feedstock in biology. The catalytic rate for the reduction of methyl viologen with **2**-**ImC**_**60**_ is similar to that observed when using a covalently appended zinc porphyrin-C_60_ dyad as the photoredox catalyst in which the porphyrin is directly excited^52^. On the other hand, the photoredox catalytic first-order rate constant becomes *k*=5.7 × 10^−8^±0.2% s^−1^ in **1**-**ImC**_**60**_, and catalytic turnover is indistinguishable from the background absorption for **3**-**ImC**_**60**_ (Fig. 4a, Supplementary Figs 34–37).

The allosteric effectors employed to shuttle through (**1–3**)-**ImC**_**60**_ (one drop of acetonitrile or 2 equiv. of chloride) are remarkably mild, suggesting that they could be introduced in a photoredox catalytic mixture to address the activity of the catalyst without appreciably affecting the intrinsic properties of the reactants themselves. We thus studied the allosteric regulation of the photoredox activity of (**1–3**)**-ImC**_**60**_ *in situ*. Towards this end, a 10-μM solution of **1-ImC**_**60**_ was prepared in the presence of methyl viologen and 1-benzyl-1,4-dihydronicotinamide mixture and a number of coordination transformations were performed in the presence of an excitation source. The diminished photoredox activity displayed by **1-ImC**_**60**_ is significantly enhanced upon the addition of a single drop of acetonitrile to form **2-ImC**_**60**_, resulting in a first-order catalytic rate constant of *k*=5.4 × 10^−7^±0.1% s^−1^ (Fig. 4b, Supplementary Fig. 38). Whereas acetonitrile cannot be abstracted without evacuating the reaction mixture, the catalyst can be deactivated and reactivated *in situ* via the addition and abstraction of chloride, and thus two cycles of catalyst activation can be achieved. The reactivated catalyst displays a catalytic rate constant of *k*=5.2 × 10^−7^±0.3% s^−1^, suggesting that the light-harvesting framework can be regenerated into its active state reversibly without any significant loss in catalytic activity. ^31^P[^1^H] NMR measurements of the catalytic sample revealed no degradation of the complex throughout the two cycles. However, degradation via oxidation, detected via the characteristic ^31^P[^1^H] NMR shift of alkyldiphenylphosphine oxide at *δ*: 29 ppm, was observed upon undergoing multiple cycles of allosteric catalyst activation and deactivation.

## Discussion

In the case of Photosystem II, regulation of energy transfer from light-harvesting antenna is possible since quenching via charge transfer between excited antennae occurs in the picosecond to sub-picosecond time scale^2^, kinetically outcompeting other decay pathways that lead to photosynthetic activity. This regulatory strategy provides the basis for switchability in the inorganic system described herein; one can chemically shuttle between two electron transfer processes, one that results in quenching and one that gives rise to catalytic activity, because the former is preceded by an inherently slower energy transfer step. Furthermore, while the charge-separated state that results from quenching the antenna can in principle reduce methyl viologen, the use of a sacrificial reductant whose oxidation potential is significantly higher than that of Rh(I) prevents its catalytic turnover in the inactive state. The multi-state switch (**1–3**)**-ImC**_**60**_ directly borrows the regulatory strategies seen in nature and combines them with allosteric regulation strategies by targeting a fast electron transfer process that is triggered through coupled electronic and conformational changes. As a result, (**1–3**)**-ImC**_**60**_ achieves a bulk switching ratio between the active and inactive states of up to 39-fold in terms of energy transfer efficiency, not observed by any previously developed regulatory strategy. The fact that regulation only occurs at the antenna level in (**1–3**)**-ImC**_**60**_ suggests that this fundamental approach can be readily applied for controlling other light-harvesting and bioinspired photoredox systems.

In addition to demonstrating for the first time that the photoredox catalytic activity of a known light-harvesting, reaction centre mimic can be regulated *in situ* and reversibly, our approach to modulating electron transfer reactions allosterically also shows that coordination-based catalyst switches can be endowed with substrate selectivity. This can become a critical component of WLA catalytic switches, which have been used in the context of PCR-like inorganic substrate amplification, but which are triggered without any input selectivity. In the WLA system described herein, we have demonstrated that the activation of a central catalytic moiety in (**1–3**)**-ImC**_**60**_ only occurs when recognition of a targeted analyte provides the proper Rh(I) oxidation potential for light harvesting to occur. This degree of analyte selectivity will become important when applying the principles outlined herein to develop photoredox catalyst-based signal amplification systems and allosterically controlled photoredox catalysts. Furthermore, (**1–3**)**-ImC**_**60**_ may be coupled to nucleophile-releasing redox reactions^54^ to trigger negative feedback loops that chemically limit the efficiency of light harvesting, representing an important step towards self-regulated inorganic systems.

## Methods

### General methods

All reactions, transformations, characterization and catalytic experiments were performed in strictly oxygen- and water-free conditions inside a nitrogen-atmosphere glove box or in sealed containers under a stream of argon. All glassware, cuvettes and cells were oven-dried for 24 h. Dichloromethane (CH_2_Cl_2_), ether, toluene, acetonitrile and tetrahydrofuran (THF) solvents were transferred from an oxygen- and water-free solvent system (J.C. Meyer) into sealed containers and purged with a stream of argon for 20 min before use. All the other solvents were purchased as HPLC grade, similarly purged for 20 min with argon and stored with activated molecular sieves. Deuterated solvents were purchased from Cambridge Isotope Laboratories and were degassed with a stream of argon before use. 5,15-Bis(mesityl)-10,20-bis(4-(chloro-methyl)phenyl)porphyrin]zinc^55^, **ImC**_**60**_^50^, P,*N*-Bodipy (**5**), P,*S*-benzyl (**S4**), model complexes **S1** and **S2**^40^, and methlyviologen hexafluorophosphate^56^ were prepared from literature procedures. 1-benzyl-1,4-dihydronicotineamide was purchased from TCI chemicals and was used as received. All other chemicals were purchased from Aldrich Chemical Co. and used as received. NMR spectra were recorded on a Bruker Avance 400 MHz (Supplementary Figs 39–62). ^1^H and ^13^C[^1^H] NMR spectra were referenced to residual proton and carbon resonances in the deuterated solvents. ^13^C[^1^H] NMR spectra of complexes **1**–**3** are not provided as WLA complexes give rise to markedly broad and featureless resonances (ref. 15). ^31^P[^1^H] NMR spectra were referenced to an 85% H_3_PO_4_ aqueous solution. ^19^F NMR spectra were referenced to a CFCl_3_ sample in CDCl_3_ solution. ^11^B[^1^H] NMR spectra were referenced to neat BF_3_·OEt_2_. All the chemical shifts are reported in p.p.m. High-resolution electrospray ionization (ESI) mass spectra measurements were recorded on an Agilent 6120 LC-TOF instrument in positive ion mode.

Ultraviolet–visible absorption measurements were performed in a Varian Cary 50 Bio spectrophotometer utilizing screw-cap 10-mm cell-path quartz cuvettes (VWR). Steady-state fluorescence emission measurements were carried out in a Horiba Jovin-Yvonne Fluorolog fluorimeter and fluorescence quantum yields were derived from the comparative method using fluorescein isothiocyanate in ethanol as standard. In particular, quantum yields were calculated from the least-squares fit of the integrated fluorescence emission versus absorbance values curves comprising seven dilution samples. Cyclic voltammetry measurements were performed with an Epsilon BASi potentiostat in an air-tight cell comprising a glassy carbon working electrode, a Ag wire pseudoreference electrode and a Pt wire auxiliary electrode. Samples were prepared as 5 mM solutions in 0.1 M n-Bu_4_NPF_6_ in CH_2_Cl_2_. Scan rates of 100 mV s^−1^ were used in all measurements. All cyclic voltammetry graphs show voltages versus the ferrocene/ferrocenium couple. Titrations were performed via the addition of aliquots from a 1 mM dichlorobenzene solution into a 1 μM CH_2_Cl_2_ solution of Rh(I) coordination complex.

Titrations of **ImC**_**60**_ were studied via ultraviolet–visible spectroscopy at room temperature in a screw-cap cuvette with a total volume of 2 ml and a constant hosts **1–3** concentration of 1 μM in CH_2_Cl_2_. Samples were prepared in a glove box. Titrations were performed via the addition of aliquots of 1 mM **ImC**_**60**_ solution in 1,2-dichlorobenzene into the solution of hosts **1–3**. Binding affinities (*K*_a_) were calculated by monitoring the change in absorbance at 434 nm, corresponding to the axially coordinated porphyrin Soret band. Binding affinity was obtained by non-linear regression analysis of the binding curves utilizing equation (1):


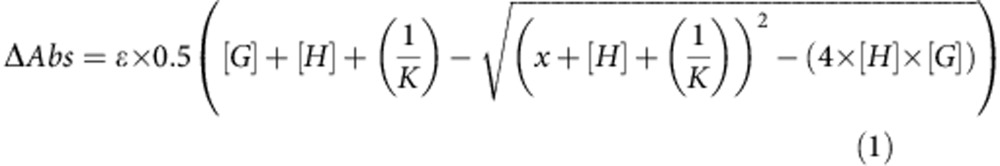


### Photoredox catalysis

Catalytic experiments were performed in screw-cap 10-mm cell-path quartz cuvettes containing 0.1 mM methlyviologen hexafluorophosphate and 0.1 mM 1-benzyl-1,4-dihydronicotineamide in CH_2_Cl_2_. Methyl viologen was added from a concentrated methanolic solution. **ImC**_**60**_ was added from a concentrated 1,2-dichlorobenzene solution. In a typical experiment, the reagents, catalyst, **ImC**_**60**_ and a stir bar were loaded inside the cuvette in the dark inside a glove box. The cuvette was placed inside a ultraviolet–visible spectrophotometer and the solution was vigorously stirred with a magnetic stirrer. An encasing was built above the cuvette holder to provide a stream of argon while performing the measurements. A laser diode (PicoQuant) was placed inside the spectrophotometer perpendicular to the probe beam and excitation power was monitored regularly using a S130C slim photodiode power sensor connected to PM200 power and energy metre console (Thorlabs). The redox reaction was monitored by tracking the absorbance changes at 630 nm, characteristic of reduced methyl viologen species. To add allosteric effectors to the reaction mixture, excitation was halted and the cuvette was taken into a glove box and then returned to the spectrophotometer.

Experimental catalytic rate constants (*k*) were obtained by non-linear regression analysis of the kinetic curves produced by plotting the product concentration (derived from Beer’s Law) versus time. Equation [*P*]=R_0_(1-e^−kt^) was fit to the experimental value using the program GraphPad using an initial starting material concentration of 0.1 M.

### Time-resolved optical spectroscopy

Visible/near-infrared femtosecond TA spectroscopy was performed on an instrument that is described elsewhere^57^. In brief, the 827-nm fundamental output of a commercial Ti:sapphire laser system (Tsunami oscillator / Spitfire amplifier, Spectra-Physics) was frequency doubled to 414 nm (~150 μJ per pulse). In our previous study, this was attenuated and used as the pump; here it is used to pump a seeded, two-stage, laboratory-constructed optical parametric amplifier. The signal output of the optical parametric amplifier was tuned to the desired wavelength then attenuated and chopped at 500 Hz. The single-filament continuum probe was generated by focusing into the appropriate non-linear medium for the desired probe range. For visible continuum (360–800 nm), ~1.5 μJ per pulse was tightly focused into a 5-mm cuvette containing a 1:1 mixture of H_2_O:D_2_O, while for the NIR probe (800–1,600 nm), ~5 μJ per pulse was loosely focused into a proprietary source (Ultrafast Systems, LLC). The residual fundamental was filtered using an appropriate edge filter. The polarization of the pump beam was rotated to 54.7° (‘magic’ angle) relative to the horizontal probe. Both pump and probe were focused to ~100 μm at the sample. After interaction, the transmitted probe was then coupled into an optical fibre and detected using the appropriate detector (customized Helios, Ultrafast Systems, LLC).

TA signals were fit to a convolution of a Gaussian instrument response function with the sum of a multi-exponential decay and a step function for signals extending far beyond the experimental window. Uncertainties are reported as the standard error of the fit.

### X-ray crystallography

Single crystals were mounted using oil (Infineum V8512) on a glass fibre. All measurements were made on a CCD area detector with MX Optics Cu Kα radiation. Data were collected using Bruker APEXII detector and processed using APEX2 from Bruker. All the structures were solved by direct methods and expanded using Fourier techniques. The non-hydrogen atoms were refined anisotropically. Hydrogen atoms were included in idealized positions, but not refined. Crystallographic information is provided in Supplementary Table 3 and Supplementary Data 1.

### Synthesis of P,S-Bz-ZnP(mes)_2_-Bz-S,P (4)

5,15-Bis(mesityl)-10,20-Bis(4-(chloro-methyl)phenyl)porphyrin]zinc **S5** (1.50 g, 1.75 mmol) and P,S-TIPS **S6** (1.48 g, 3.67 mmol) were dissolved in 100 ml of degassed THF in a Schlenk flask. 1.52 g of CsF were added and the mixture was left to stir in the dark at 60 °C for 24 h under a nitrogen atmosphere. The reaction mixture was filtered and the solvent was evacuated. The resulting solid was redissolved in a small amount of CH_2_Cl_2_ and poured onto a large silica plug. The P,S-TIPS was washed with excess CH_2_Cl_2_ and a deep purple band was eluted with a 1:1 mixture of CH_2_Cl_2_:THF. The product was isolated, redissolved in hot CH_2_Cl_2_ with a minimal amount of THF and crashed out solution by adding pentane, affording **4** as deep purple needles (1.92 g, 75% yield). ^1^H NMR (400.16 MHz, 25 °C, CD_2_Cl_2_): *δ* 8.71 (dd, *J*_H-H_=6 Hz, 8 H), 7.92 (d, *J*_H-H_=8 Hz, 4 H), 7.37 (d, *J*_H-H_=8 Hz, 4 H), 7.30 (s, 4 H), 7.13 (m, 12 H), 6.58 (m, 8 H), 3.77 (s, 4 H), 2.63 (s, 6 H), 1.77 (m, 16 H), 1.60 (m, 4 H). ^13^C[^1^H] (100.63 MHz, 25 °C, CD_2_Cl_2_): *δ* 150.3 (s), 142.0 (s), 140.3 (s), 139.6 (s), 138.5 (d, J_C-P_=12 Hz), 137.9 (s), 136.3 (s), 135.0 (s), 133.1 (s), 132.7 (s), 132.4 (s), 130.9 (s), 129.1 (s), 128.8 (d, *J*_C-P_=5 Hz), 128.1 (s), 127.3 (s), 119.9 (s), 119.1 (s), 36.6 (s), 28.7 (d, *J*_C-P_=17 Hz), 27.2 (d, *J*_C-P_=15 Hz), 22.9 (s), 21.6 (s). ^31^P[^1^H] NMR (161.98 MHz, 25 °C, CD_2_Cl_2_): *δ* -19.1 (s). HRMS (ESI+) *m/z* calculated for [M]^+^: 1276.3809; found: 1276.3802.

### Synthesis of [Rh_2_(κ_2_:η_2_:κ_2_-4)(κ_2_-5)_2_](OTf)_2_ (1)

Rh_2_Cl_2_(cyclooctene)_4_ (3.6 mg, 0.010 mmol) was dissolved in 3 ml of a 1:1 solution of CH_2_Cl_2_:THF and 3 ml of **4** (6.4 mg, 0.005 mmol) in THF were added dropwise over the course of 5 min. Immediately afterwards, **3** (5.3 mg, 0.010 mmol) dissolved in 3 ml of THF was added dropwise. The solution was sonicated for 10 min and was stirred for 3 h. The solution was concentrated to about 1 and 4 ml of CH_2_Cl_2_ were added. Tl(OTf; 3.6 mg, 0.010 mmol) was added and the mixture was stirred for 1 h and filtrated through a pad of Celite. The product was recrystallized from a CH_2_Cl_2_/pentane solution twice, yielding 1 as a dark red solid (*in situ*^31^P[^1^H]} NMR yields=90–95%, isolated yield 12.1 mg, 85%). ^1^H NMR (400.16 MHz, 25 °C, CD_2_Cl_2_): *δ* 7.61 (d, *J*_H-H_=4 Hz, 2 H), 7.45–7.15 (m, 15 H), 7.12–7.00 (m, 8 H), 5.90 (m, 1 H), 4.26 (s, 2 H), 3.91 (m, 2H), 2.53 (m, 2H), 2.38 (m, 10 H), 2.20 (s, 6 H), 2.12–1.95 (m, 4 H), 1.02 (t, *J*_H-H_=8 Hz, 6 H). ^31^P[^1^H] NMR (161.98 MHz, 25 °C, CD_2_Cl_2_): *δ* 66.8 (dd, *J*_P-P_=38 Hz, *J*_P-Rh_=178 Hz, 2P), 50.0 (dd, *J*_P-P_=38 Hz, *J*_P-Rh_=166 Hz, 2P). ^19^F NMR (376.49 MHz, 25 °C, CD_2_Cl_2_): *δ* −79.4 (s, 6F), −145.6 (m, 4F). ^11^B[^1^H] NMR (128.38 MHz, 25 °C, CD_2_Cl_2_): *δ* 0.09 (t, *J*_B-F_=32 Hz). HRMS (ESI+) *m/z* calculated for [M-2OTf]^2+^: 1272.3746; found: 1272.3762.

### Synthesis of [Rh_2_(CH_3_CN)_2_(κ_2_:η_2_:κ_2_-4)(5)_2_](OTf)_2_ (2)

Complex **1** (5 mg, 1.8 × 10^−3^ mmol) was dissolved in 1 ml of CH_2_Cl_2_ and one drop of acetonitrile was added (^31^P[^1^H] NMR yield: quantitative). ^1^H NMR (400.16 MHz, 25 °C, CD_2_Cl_2_): *δ* 7.61 (d, *J*_H-H_=4 Hz, 2 H), 7.45–7.15 (m, 15 H), 7.12–7.00 (m, 8 H), 5.90 (m, 1 H), 4.26 (s, 2 H), 3.91 (m, 2H), 2.53 (m, 2H), 2.38 (m, 10 H), 2.20 (s, 6 H), 2.12–1.95 (m, 4 H), 1.02 (t, *J*_H-H_=8 Hz, 6 H). ^31^P[^1^H] NMR (161.98 MHz, 25 °C, CD_2_Cl_2_): *δ* 69.2 (dd, *J*_P-P_=44 Hz, *J*_P-Rh_=172 Hz, 2P), 25.8 (dd, *J*_P-P_=44 Hz, *J*_P-Rh_=160 Hz, 2P). ^19^F NMR (376.49 MHz, 25 °C, CD_2_Cl_2_): *δ* −79.0 (s, 6F), −145.4 (q, *J*_F-B_=34 Hz, 4F). ^11^B[^1^H] NMR (128.38 MHz, 25 °C, CD_2_Cl_2_): *δ* 0.74 (t, *J*_B-F_=32 Hz). HRMS (ESI+) *m/z* calculated for [M-2OTf]^2+^: 1313.3011; found: 1313.3035.

### Synthesis of [Rh_2_Cl_2_(κ_2_:η_2_:κ_2_-4)(5)_2_] (3)

Complex **1** (5 mg, 1.8 × 10^−3^ mmol) was dissolved in 1 ml of CH_2_Cl_2_ and tetrabutylammonium chloride was added. The product was precipitated from solution via the addition of pentane (^31^P[^1^H] NMR yield: quantitative, isolated yield 4.4 mg, 96%). ^1^H NMR (400.16 MHz, 25 °C, CD_2_Cl_2_): *δ* 7.61 (d, *J*_H-H_=4 Hz, 2 H), 7.45–7.15 (m, 15 H), 7.12–7.00 (m, 8 H), 5.90 (m, 1 H), 4.26 (s, 2 H), 3.91 (m, 2H), 2.53 (m, 2H), 2.38 (m, 10 H), 2.20 (s, 6 H), 2.12–1.95 (m, 4 H), 1.02 (t, *J*_H-H_=8 Hz, 6 H). ^31^P[^1^H] NMR (161.98 MHz, 25 °C, CD_2_Cl_2_): *δ* 71.4 (dd, *J*_P-P_=40 Hz, *J*_P-Rh_=186 Hz, 2P), 30.8 (dd, *J*_P-P_=40 Hz, *J*_P-Rh_=166 Hz, 2P). ^19^F NMR (376.49 MHz, 25 °C, CD_2_Cl_2_): *δ* −145.4 (q, *J*_F-B_=34 Hz, 2F). ^11^B[^1^H] NMR (128.38 MHz, 25 °C, CD_2_Cl_2_): *δ* 0.23 (t, *J*_B-F_=33 Hz). HRMS (ESI+) *m/z* calculated for [M-2Cl]^2+^: 1272.3746; found: 1272.3760.

## Author contributions

A.M.L. and C.A.M. developed the concept, and C.A.M. supervised and guided the research. All experiments were designed and performed by A.M.L., J.M.-A. and C.M.M. A.M.L. and R.M.Y. performed all TA spectroscopy measurements under the supervision of M.R.W. C.L.S. performed all crystallographic studies. A.M.L. and C.A.M. co-wrote the manuscript. All the authors discussed the results and commented on the manuscript during its preparation.

## Additional information

**Accession codes:** The X-ray crystallographic coordinates for structures reported in this Article have been deposited at the Cambridge Crystallographic Data Centre (CCDC), under deposition numbers CCDC 1045099-1045101. These data can be obtained free of charge from The Cambridge Crystallographic Data Centre via www.ccdc.cam.ac.uk/data_request/cif.

**How to cite this article**: Lifschitz, A. M. *et al*. An allosteric photoredox catalyst inspired by photosynthetic machinery. *Nat. Commun.* 6:6541 doi: 10.1038/ncomms7541 (2015).

## Supplementary Material

Supplementary Information Supplementary Figures 1-62, Supplementary Tables 1-3, Supplementary Methods and Supplementary Reference

Supplementary Data 1 Crystallographic Information Files for compounds 4, 5 and X1.

## Figures and Tables

**Figure 1:**
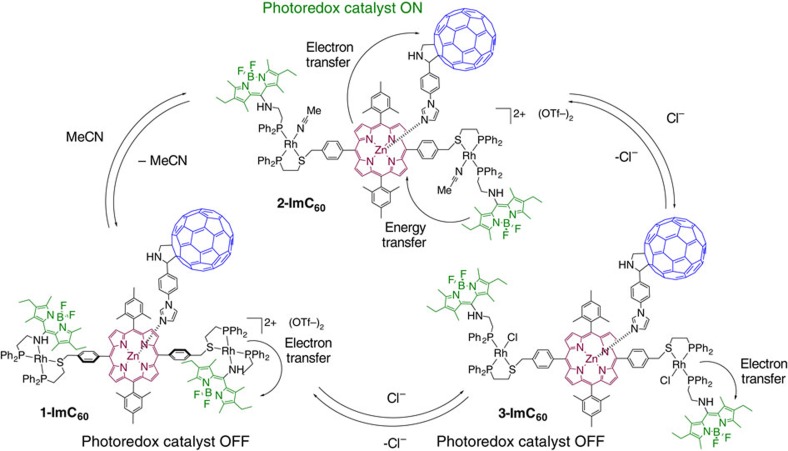
Allosteric regulation of a light-harvesting antenna/reaction centre mimic. Activation of the catalytically active reaction centre mimic on the excitation of the antenna at 480 nm is controlled via coordination chemistry at a distal, redox-active Rh(I) centre. Light-harvesting antenna is shown in green and the reaction centre is shown in magenta and blue.
