# Supplementary material for: An allosteric photoredox catalyst inspired by photosynthetic machinery
Source: Nat Commun. 2015 Mar 30;6:6541. doi: 10.1038/ncomms7541 (PMC4389231; doi:10.1038/ncomms7541)
Supplement: Supplementary Information — Supplementary Figures 1-62, Supplementary Tables 1-3, Supplementary Methods and Supplementary Reference [file ncomms7541-s1.pdf]

Supplementary Figures:

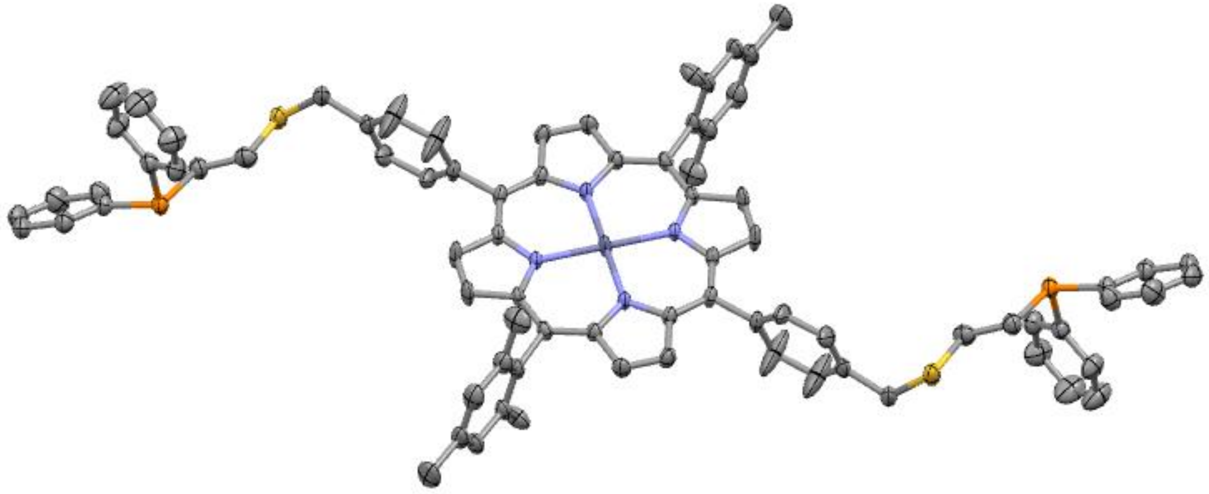

Supplementary Figure 1. Crystal structure of ligand **4** drawn with 50% thermal ellipsoid probability. Hydrogens are omitted for clarity. Zinc atoms are dark blue; sulfur, yellow; phosphorus, orange; nitrogen, blue; carbon, grey. The structure shows that the benzyl spacers introduce a kink that allows for the incorporation of **ImC$_{60}$** into **1-3** with similar binding constants regardless of changes in steric hindrance.

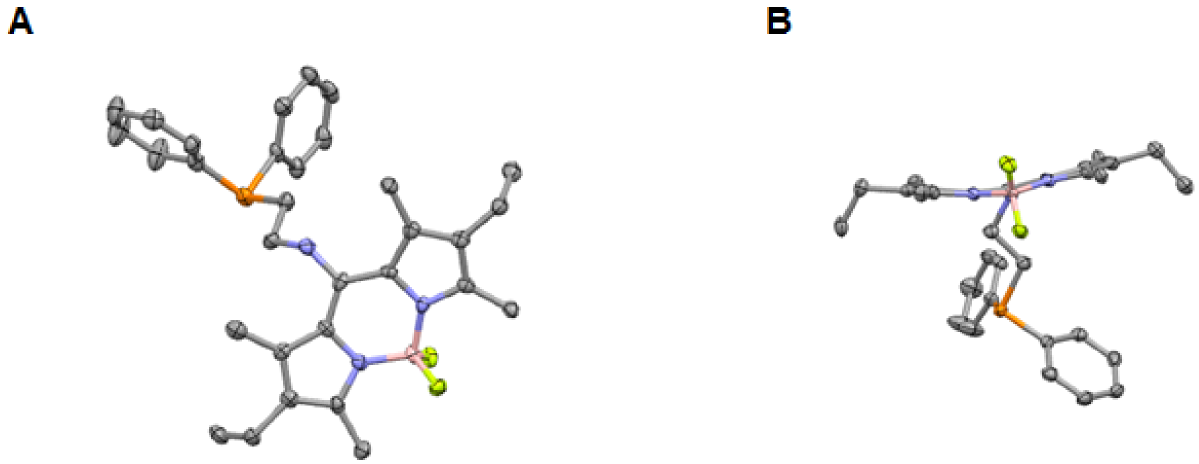

Supplementary Figure 2. Crystal structure of ligand **5** drawn with 50% thermal ellipsoid probability. Hydrogens are omitted for clarity. Phosphorus atoms are orange; nitrogen, blue; fluorine, bright yellow; carbon, grey; boron, pink. The structure shows that the nitrogen in the coordinating moiety is not electronically coupled to the Bodipy.

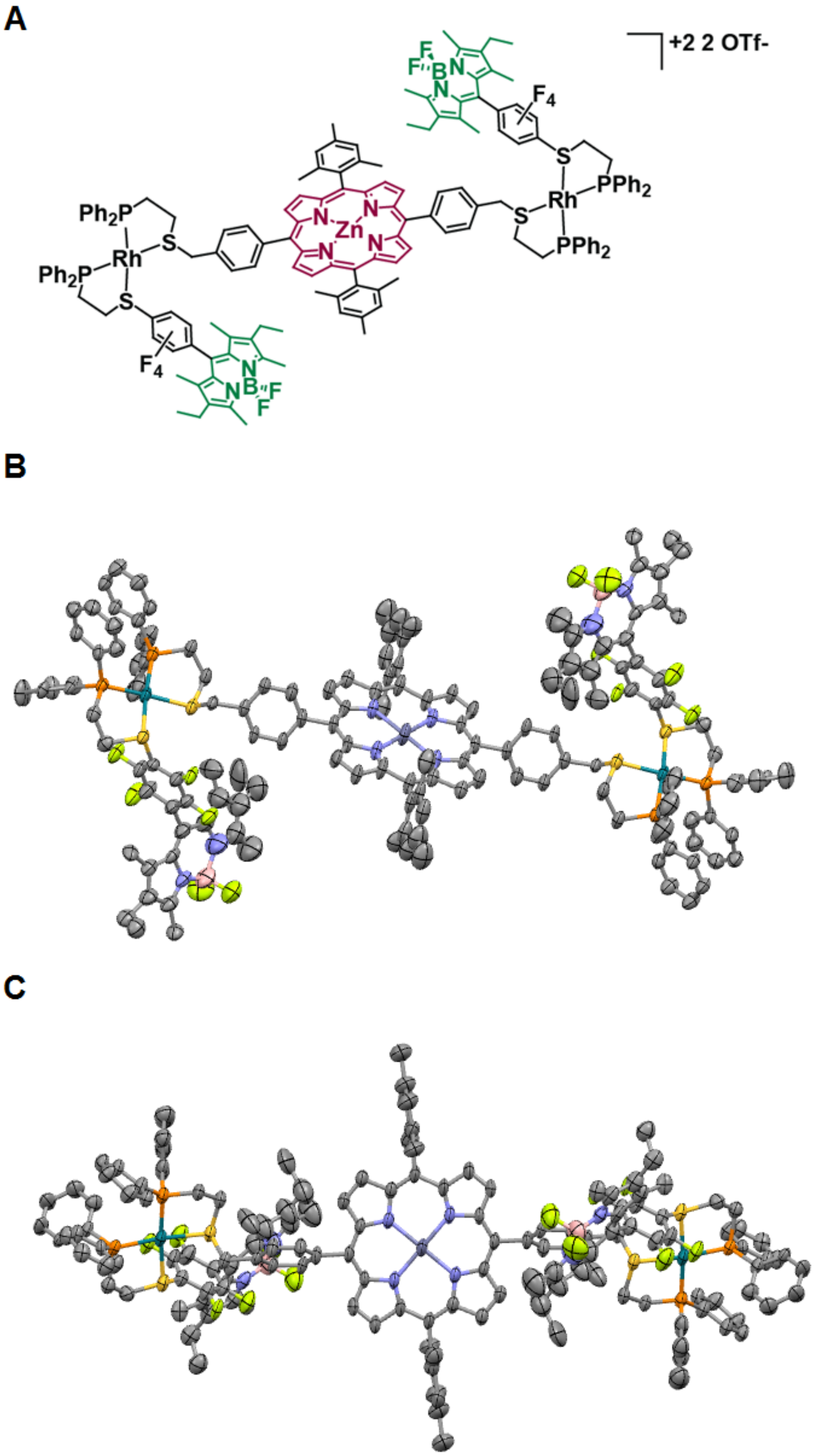

Supplementary Figure 3. While we were not able to grow single crystals of **1-3**, were able to do so for a complex analogous to **1** (A). Crystal structure drawn with 50% thermal ellipsoid probability (B). Hydrogens are omitted for clarity. Rhodium atoms are green; zinc, dark blue; sulfur, yellow; phosphorus, orange; nitrogen, blue; fluorine, bright yellow; carbon, grey; boron, pink. The top view of the structure shows that the zinc porphyrin remains exposed, allowing for axial coordination of **ImC$_{60}$**.

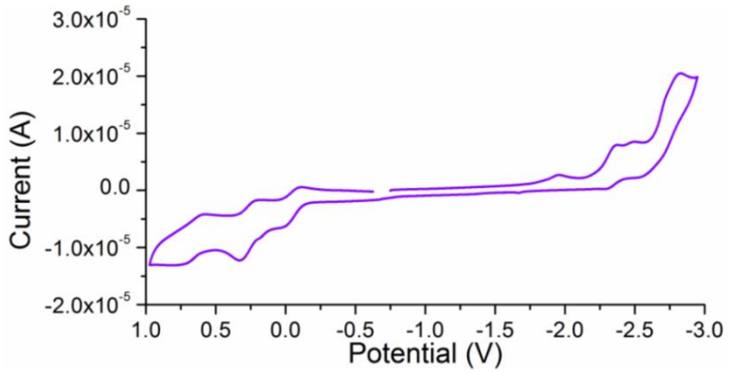

Supplementary Figure 4. Cyclic voltammograms of model complex **1** in 0.1 M $N(n\text{-}Bu)_4PF_6$ solution in CH_2Cl_2 (potential vs. ferrocene/ferrocenium, scan rate: 100 mV/s).

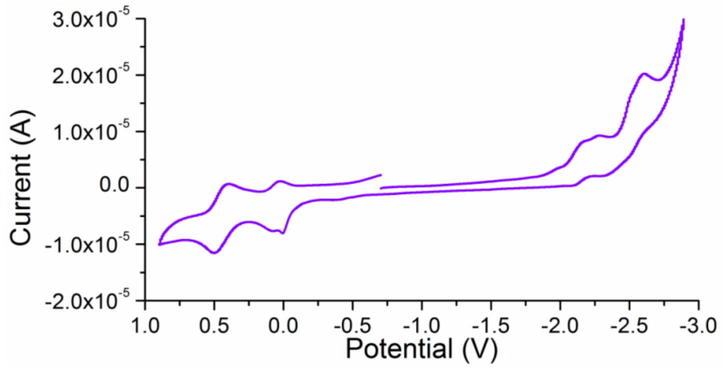

Supplementary Figure 5. Cyclic voltammograms of model complex **2** in 0.1 M $N(n\text{-}Bu)_4PF_6$ solution in CH_2Cl_2 (potential vs. ferrocene/ferrocenium, scan rate: 100 mV/s).

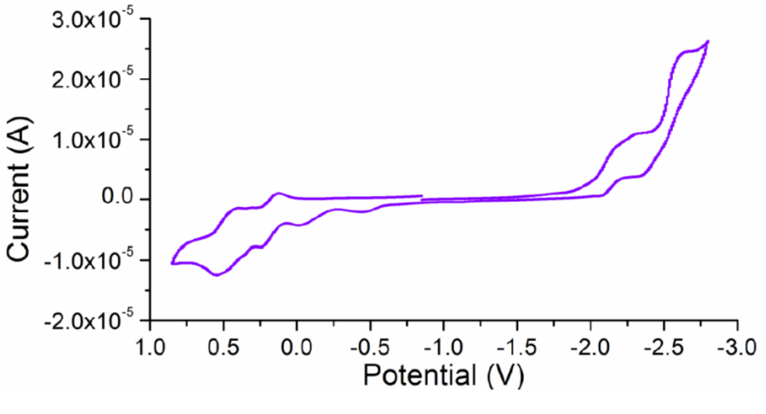

Supplementary Figure 6. Cyclic voltammograms of model complex **3** in 0.1 M $N(n\text{-}Bu)_4PF_6$ solution in CH_2Cl_2 (potential vs. ferrocene/ferrocenium, scan rate: 100 mV/s).

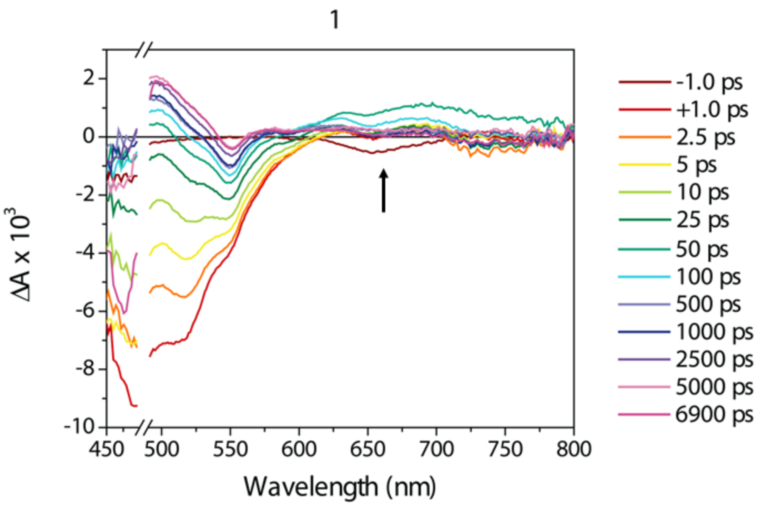

Supplementary Figure 7. Transient absorption spectra of complex **1** in CH_2Cl_2 (λ_{ex} = 477 nm). Note formation of broad Bodipy anion peak at λ_{abs} = 650 nm overlapping with excited zinc porphyrin absorption band.

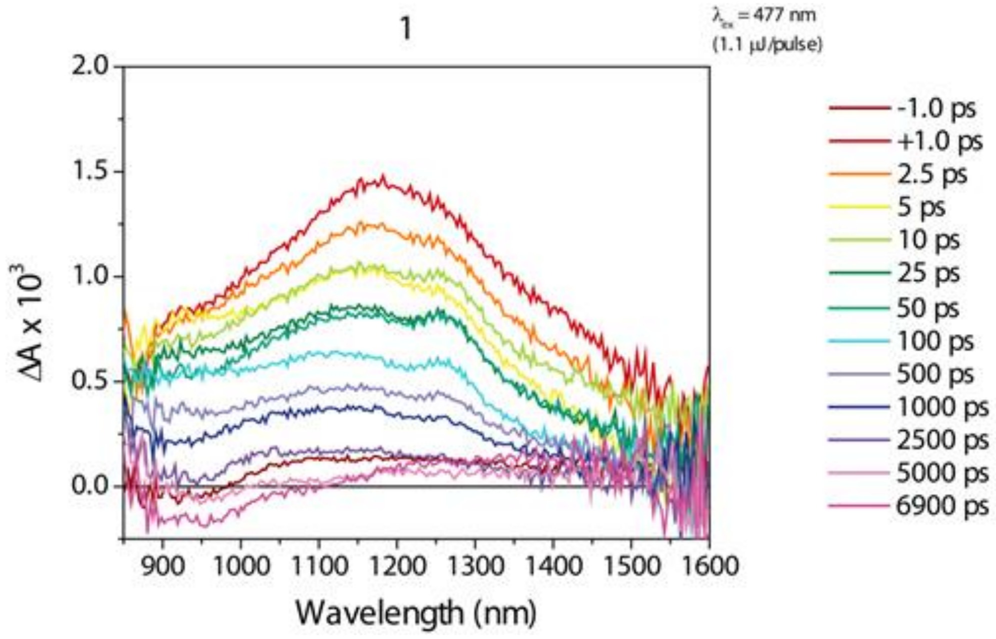

Supplementary Figure 8. NIR transient absorption spectra of complex **1** in CH_2Cl_2 (λ_{ex} = 477 nm).

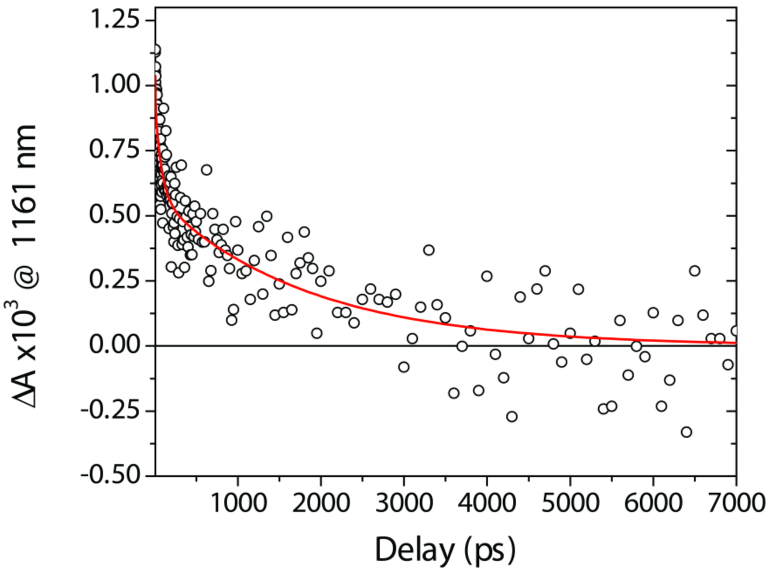

Supplementary Figure 9. Changes in transient absorption at λ_{abs} = 1161 nm, characteristic of the Bodipy excited state, following excitation of **1** at λ_{ex} = 477 nm.

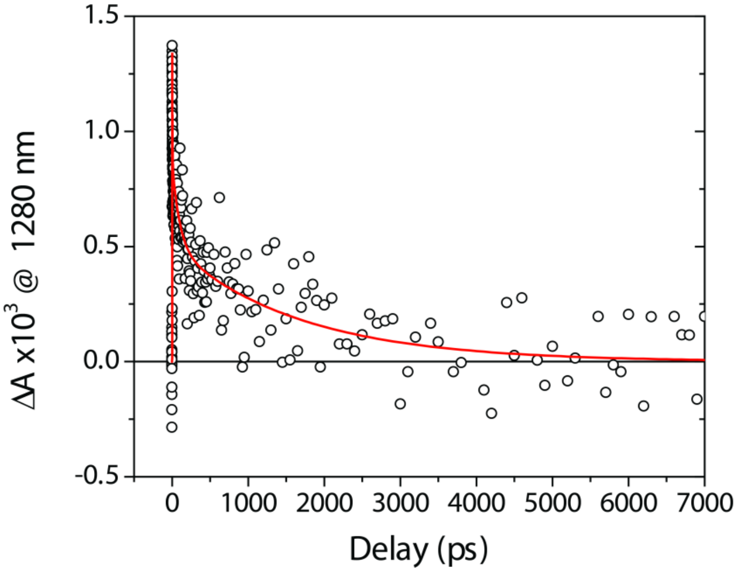

Supplementary Figure 10. Changes in transient absorption at λ_{abs} = 1280 nm, characteristic of the zinc porphyrin excited state, following excitation of **1** at λ_{ex} = 477 nm.

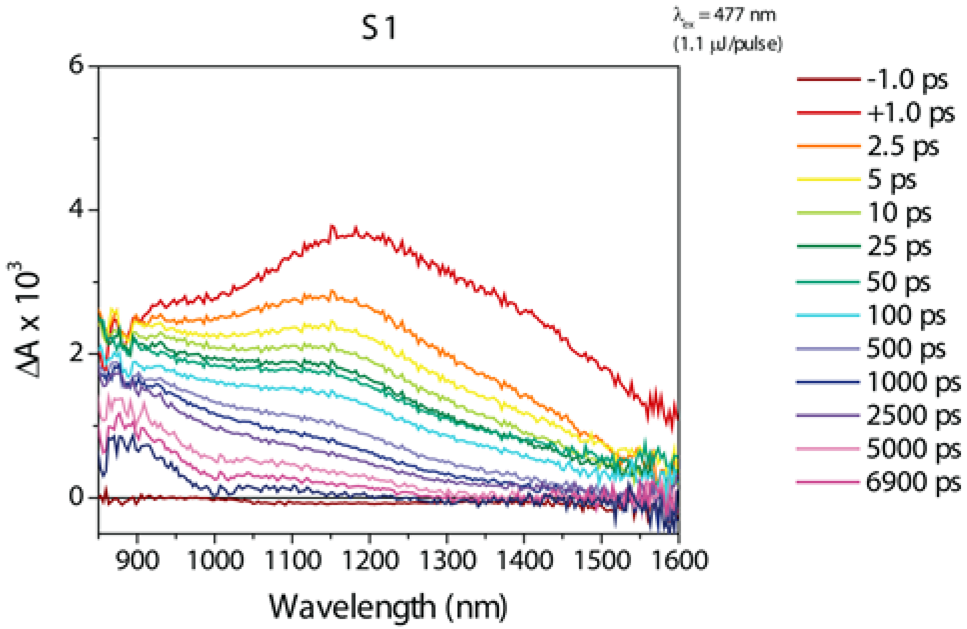

Supplementary Figure 11. NIR transient absorption spectra of complex **S1** in CH_2Cl_2 (λ_{ex} = 477 nm).

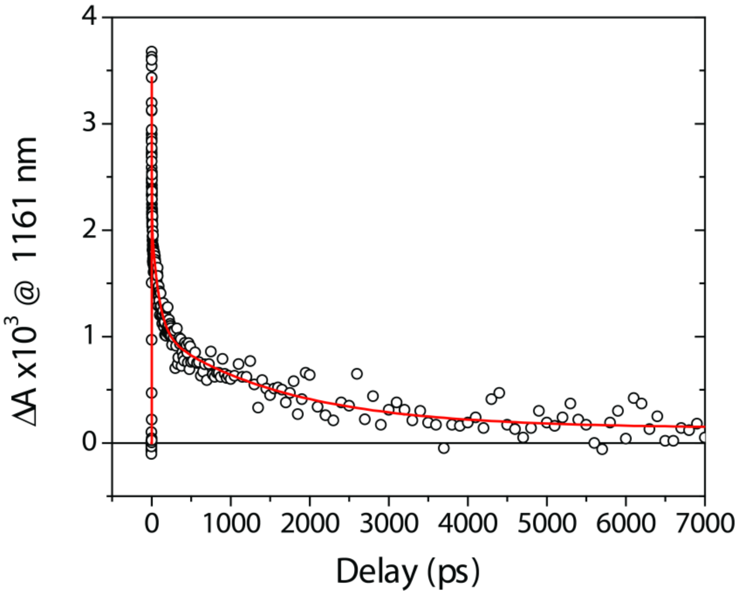

Supplementary Figure 12. Changes in transient absorption at λ_{abs} = 1161 nm, characteristic of the Bodipy excited state, following excitation of **S1** at λ_{ex} = 477 nm.

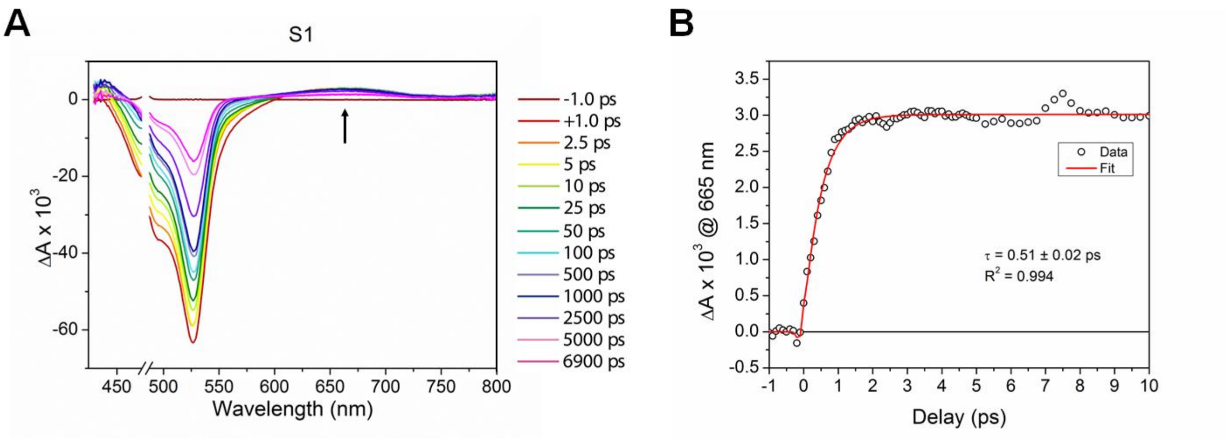

Supplementary Figure 13. Kinetics of PeT from Rh(I) to Bodipy: (A) Transient absorption spectra of complex **S1** in CH_2Cl_2 (λ_{ex} = 477 nm). (B) Changes in transient absorption at wavelengths characteristic of ground-state bleach of Bodipy (λ_{abs} = 527 nm) and the Bodipy radical anion (λ_{abs} = 660 nm) following excitation.

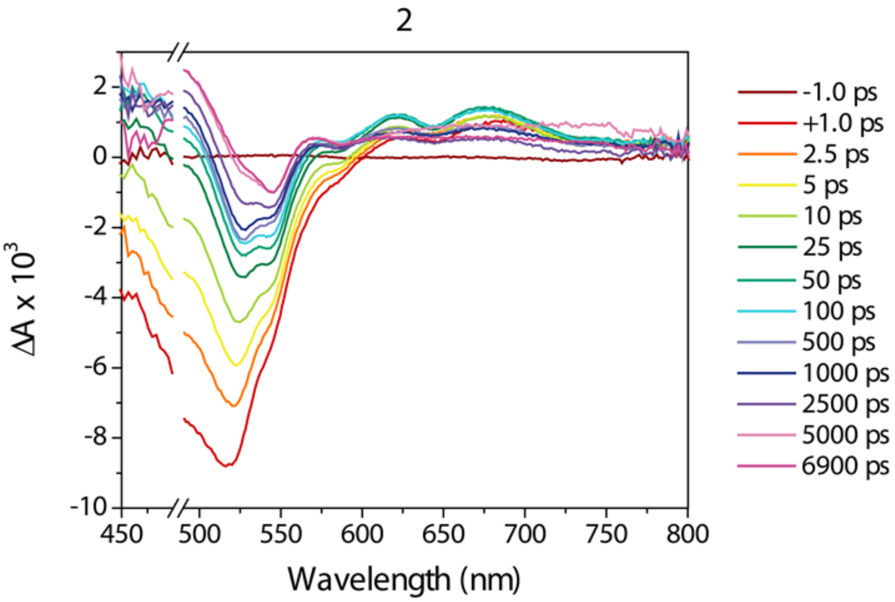

Supplementary Figure 14. Transient absorption spectra of complex **2** in CH_2Cl_2 (λ_{ex} = 477 nm).

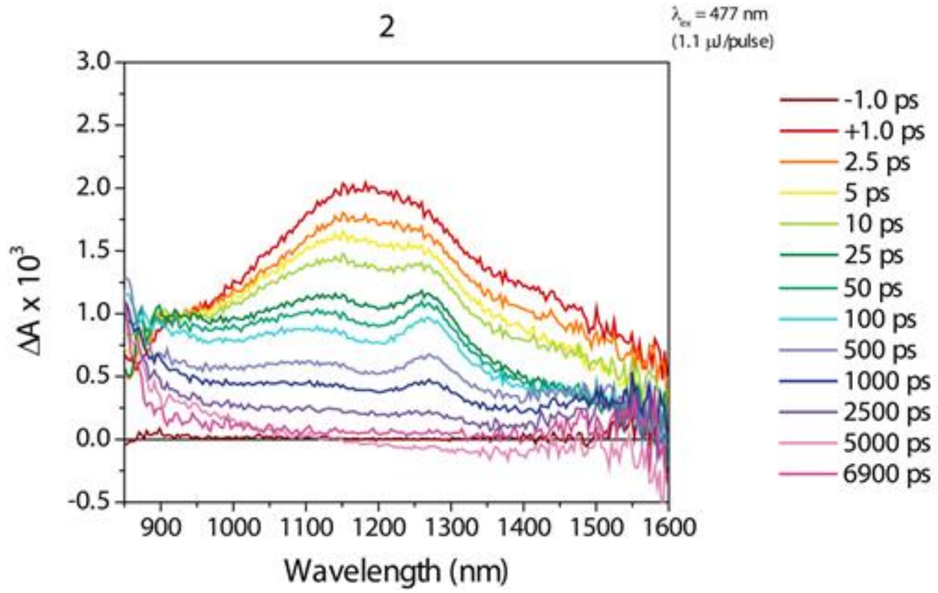

Supplementary Figure 15. NIR transient absorption spectra of complex **2** in CH_2Cl_2 (λ_{ex} = 477 nm).

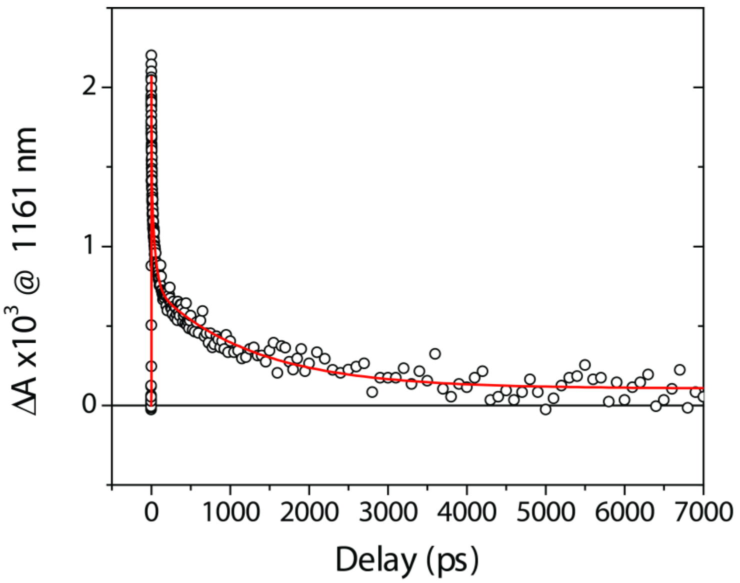

Supplementary Figure 16. Changes in transient absorption at λ_{abs} = 1161 nm, characteristic of the Bodipy excited state, following excitation of **2** at λ_{ex} = 477 nm.

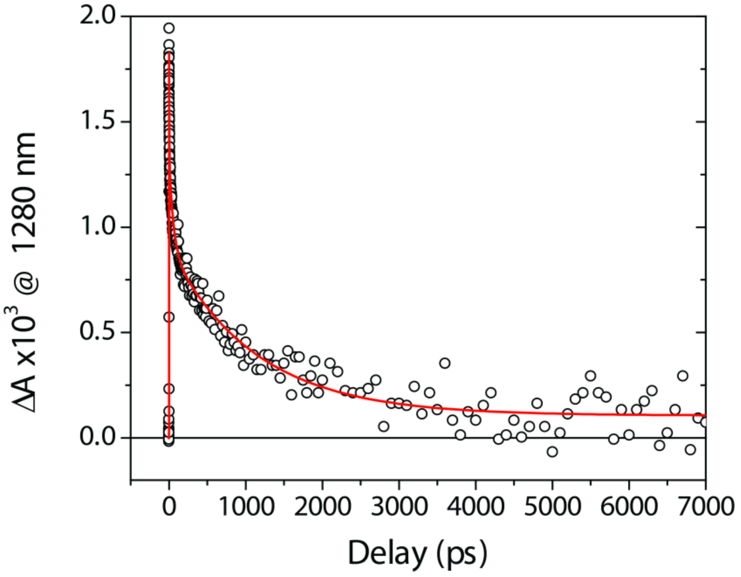

Supplementary Figure 17. Changes in transient absorption at λ_{abs} = 1280 nm, characteristic of the zinc porphyrin excited state, following excitation of **2** at λ_{ex} = 477 nm.

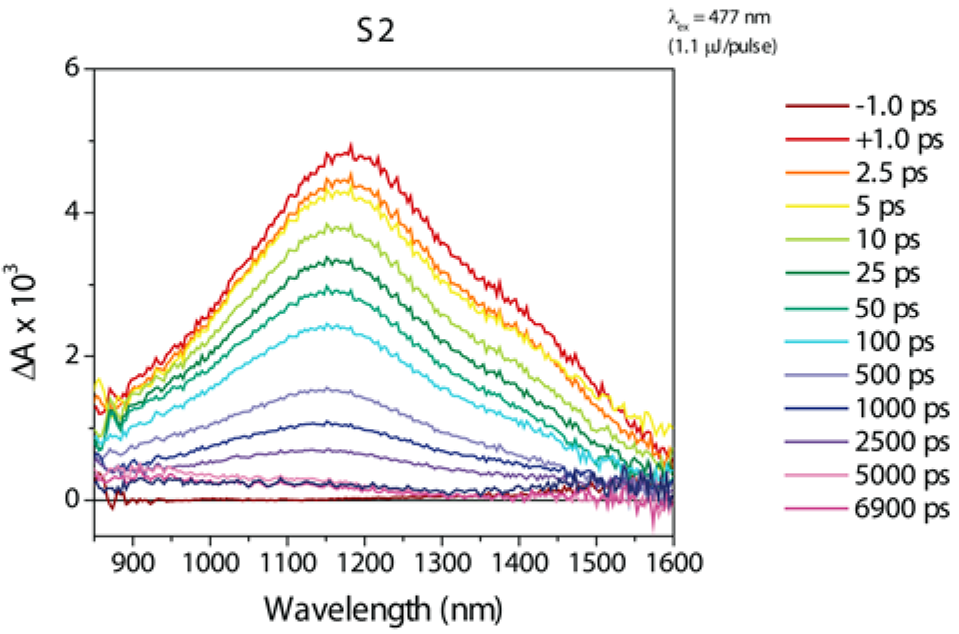

Supplementary Figure 18. NIR transient absorption spectra of complex **S2** in CH_2Cl_2 (λ_{ex} = 477 nm).

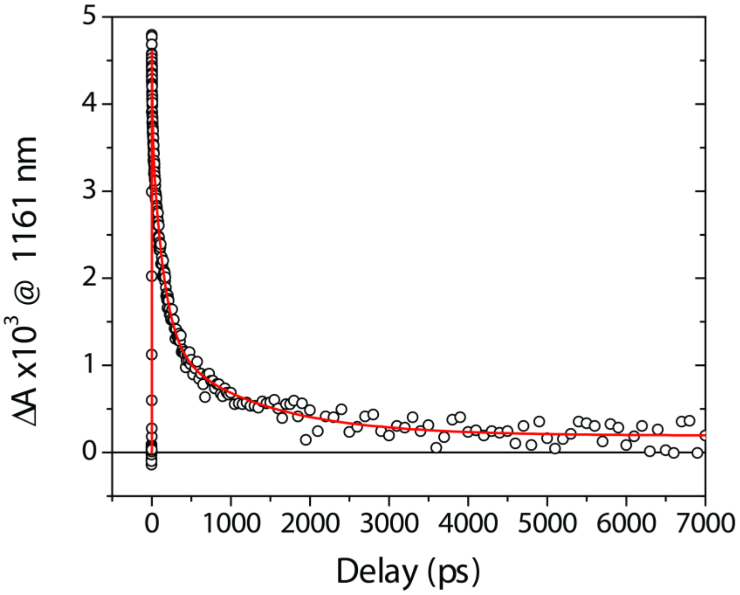

Supplementary Figure 19. Changes in transient absorption at λ_{abs} = 1161 nm, characteristic of the Bodipy excited state, following excitation of **S2** at λ_{ex} = 477 nm.

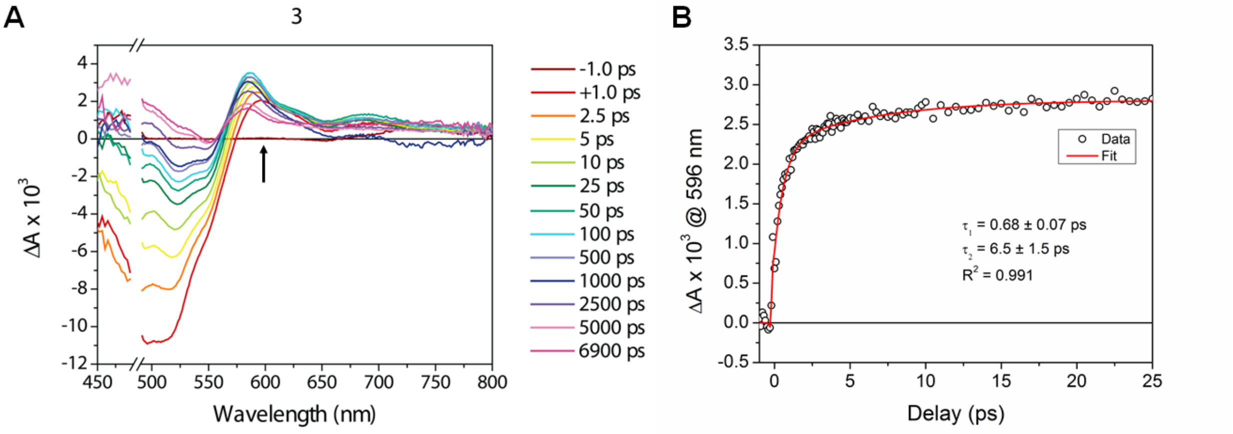

Supplementary Figure 20. Transient absorption spectra of complex **3** in CH_2Cl_2 (λ_{ex} = 477 nm) (A). Note formation of Bodipy radical anion peak at λ_{abs} = 560 nm. (B) Changes in transient absorption characteristic of the Bodipy radical anion (λ_{abs} = 596 nm) following excitation.[1]

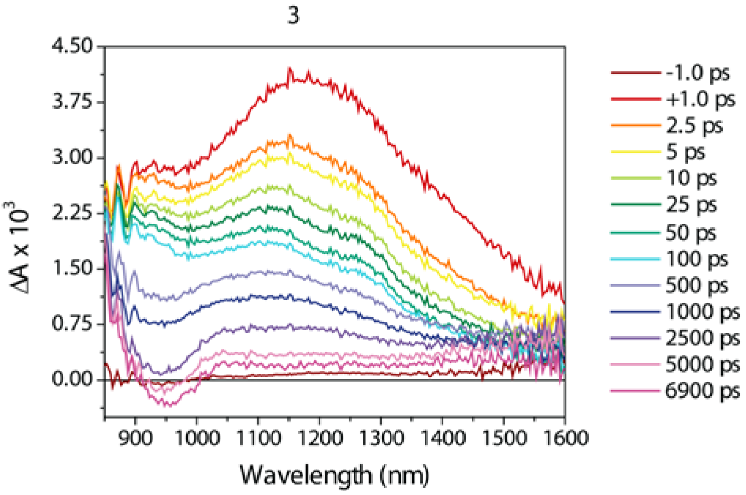

Supplementary Figure 21. NIR transient absorption spectra of complex **3** in CH_2Cl_2 (λ_{ex} = 477 nm).

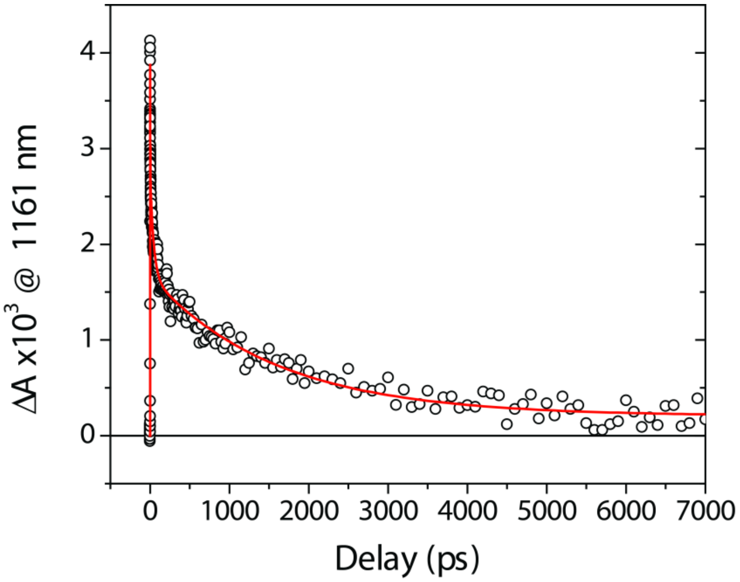

Supplementary Figure 22. Changes in transient absorption at λ_{abs} = 1161 nm, characteristic of the Bodipy excited state, following excitation of **3** at λ_{ex} = 477 nm.

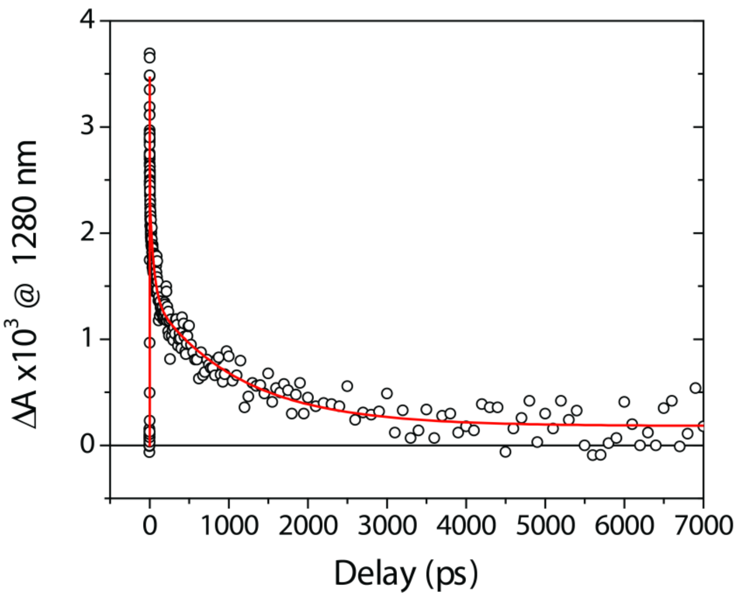

Supplementary Figure 23. Changes in transient absorption at λ_{abs} = 1280 nm, characteristic of the zinc porphyrin excited state, following excitation of **3** at λ_{ex} = 477 nm.

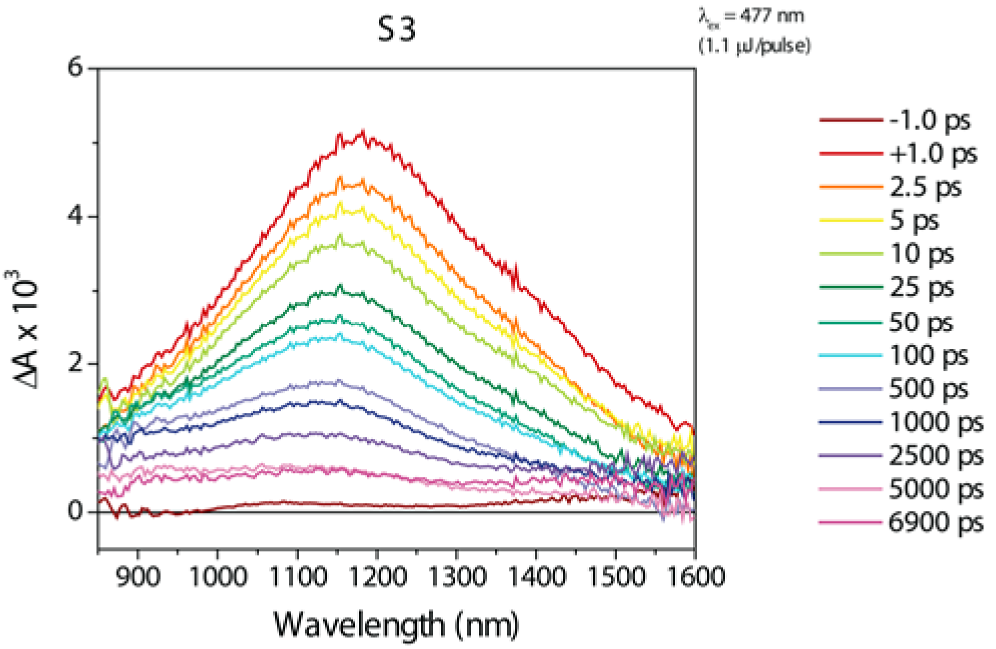

Supplementary Figure 24. NIR transient absorption spectra of complex **S3** in CH_2Cl_2 (λ_{ex} = 477 nm).

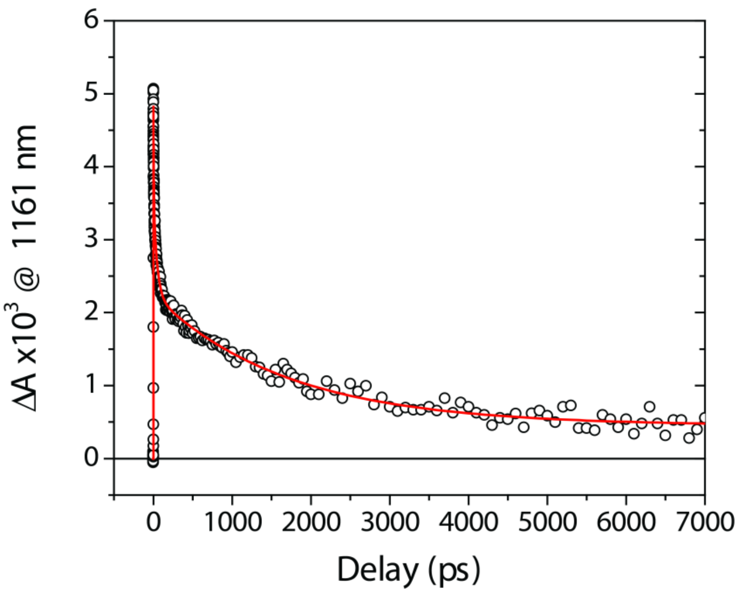

Supplementary Figure 25. Changes in transient absorption at λ_{abs} = 1161 nm, characteristic of the Bodipy excited state, following excitation of **S3** at λ_{ex} = 477 nm.

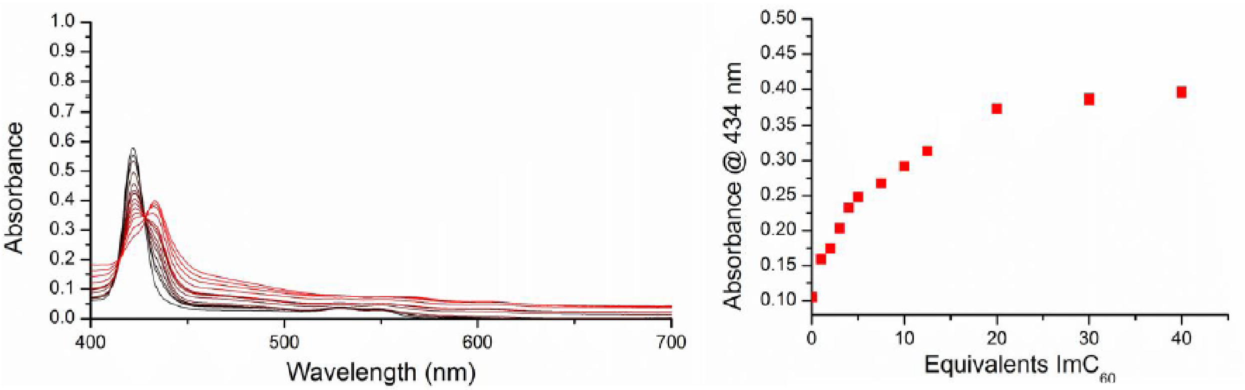

Supplementary Figure 26. UV-vis titration of ImC_{60} from a concentrated dichlorobenzene solution into a 1 µM solution of **1** in CH_2Cl_2: absorbance changes (left) and absorbance changes at 434 nm following titration (right).

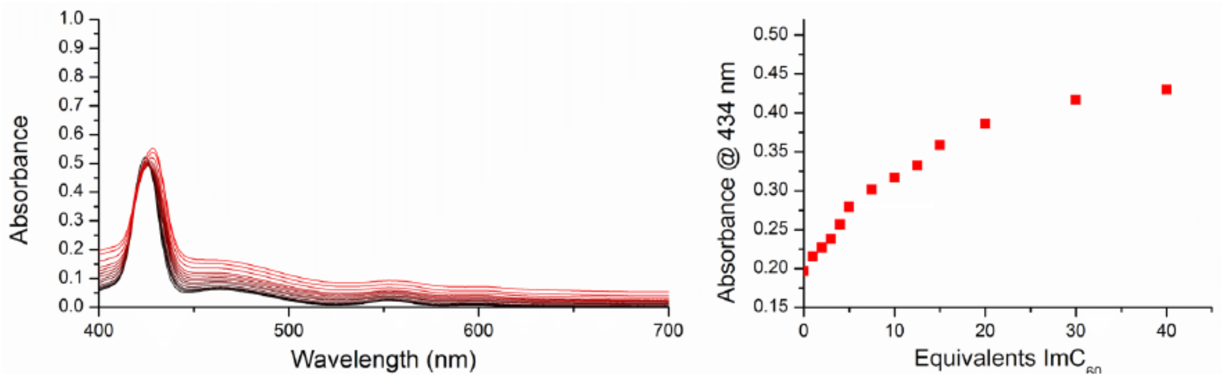

Supplementary Figure 27. UV-vis titration of ImC_{60} from a concentrated dichlorobenzene solution into a 1 µM solution of **2** in CH_2Cl_2: absorbance changes (left) and absorbance changes at 434 nm following titration (right).

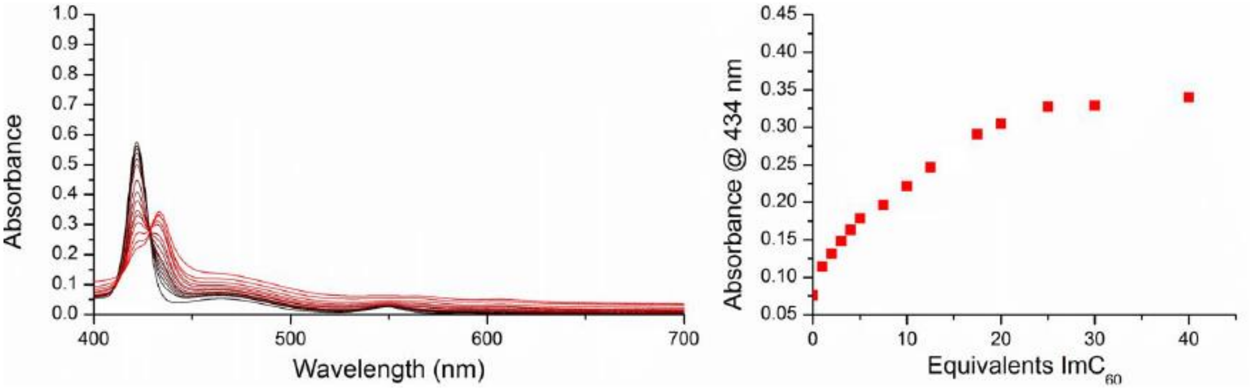

Supplementary Figure 28. UV-vis titration of ImC_{60} from a concentrated dichlorobenzene solution into a 1 µM solution of **3** in CH_2Cl_2: absorbance changes (left) and absorbance changes at 434 nm following titration (right).

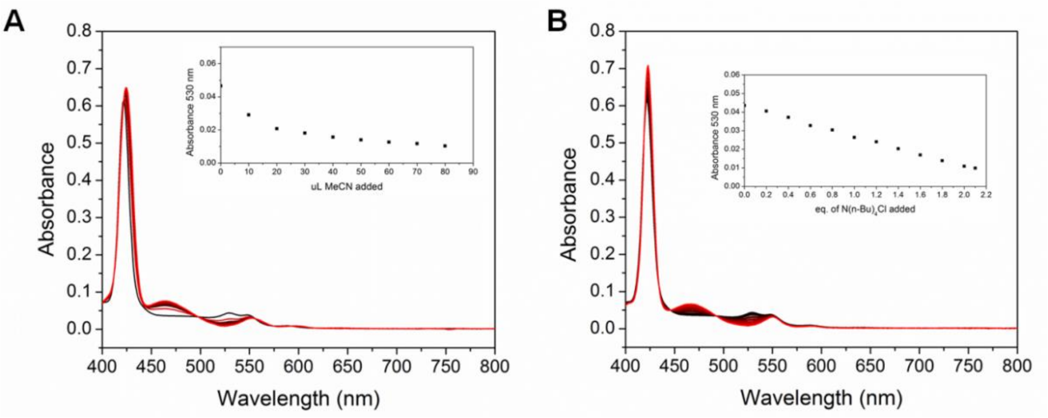

Supplementary Figure 29. UV-vis titration of allosteric effectors into a 1 µM solution of **1** in CH_2Cl_2: absorbance changes following acetonitrile (A) and $N(n\text{-}Bu)_4Cl$ addition (B).

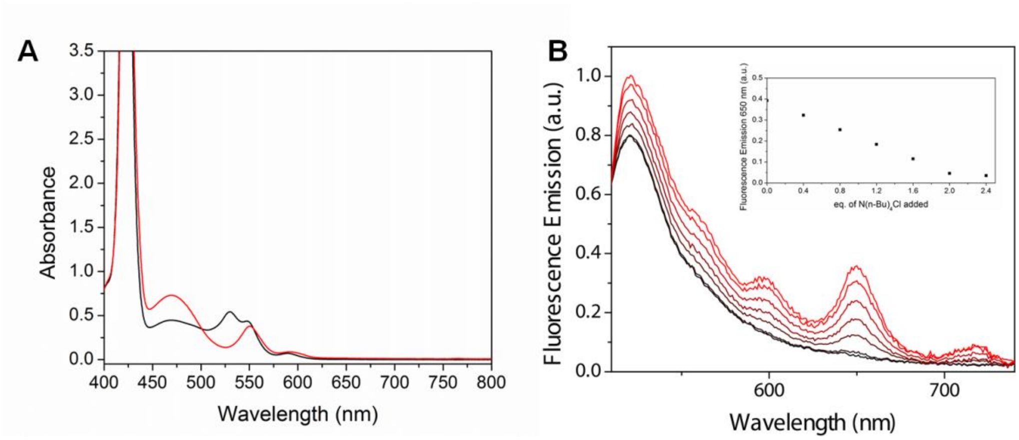

Supplementary Figure 30. UV-vis and fluorescence emission spectral changes in a 10 µM solution of **2** in CH_2Cl_2 following evacuation of the solvent mixture and redissolution in CH_2Cl_2 (A) or titration of N(n-Bu)$_4$Cl.

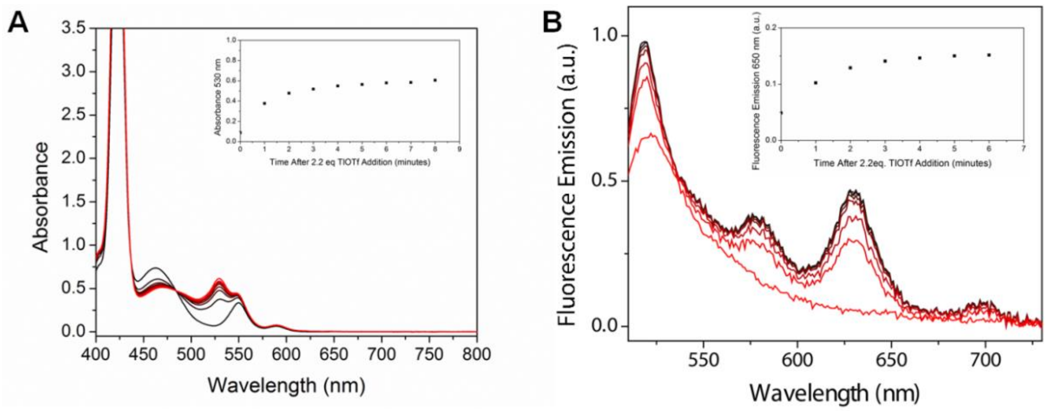

Supplementary Figure 31. UV-vis and fluorescence emission spectral changes in a 10 µM solution of **3** in CH_2Cl_2 following addition of 2.2 eq. of TlOTf in CH_2Cl_2 (A) and in CH_2Cl_2 with a drop of acetonitrile (B).

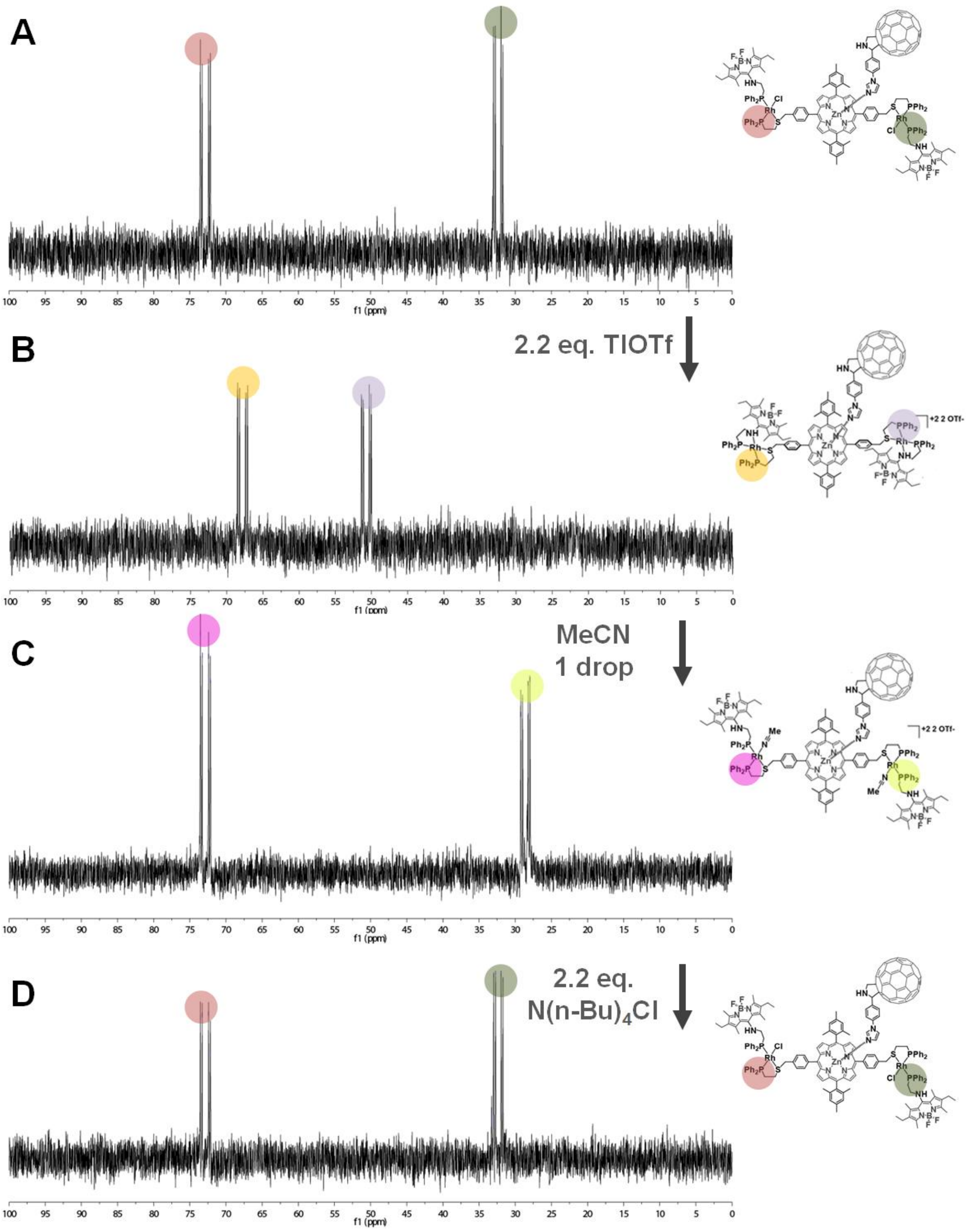

Supplementary Figure 32. In situ toggling between coordination states via allosteric inputs tracked by $^{31}P\{^{1}H\}$ NMR spectroscopy: a sample of **3-ImC$_{60}$** (A) is sequentially converted to **1-ImC$_{60}$** with 2.2 eq. of TlOTf (B), to **2-ImC$_{60}$** via the addition of a single drop of acetonitrile (C), and finally back to **3-ImC$_{60}$** via the addition of 2.2 eq. of $N(n\text{-}Bu)_4Cl$.

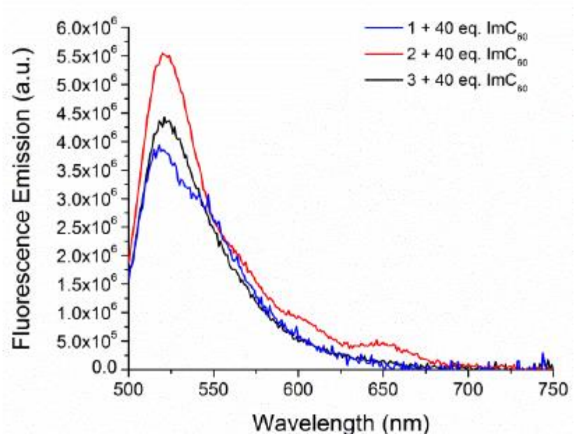

Supplementary Figure 33. Fluorescence emission spectra of 1 µM solutions of complexes **1-3** in the presence of 40 eq. of **ImC$_{60}$** (CH_2Cl_2, λ_{ex} = 480 nm).

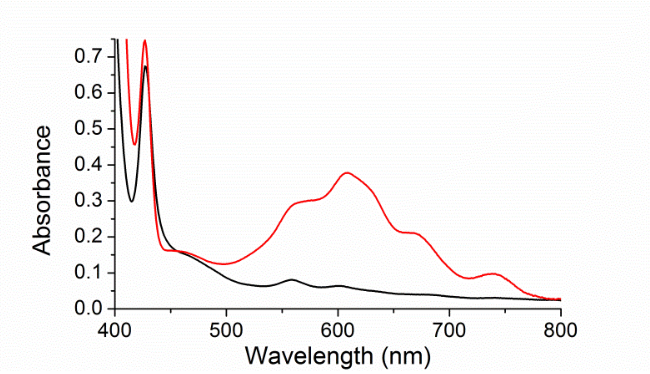

Supplementary Figure 34. Catalytic reduction of methyl viologen in the presence of 1 µM **2** and 10 eq. of **ImC$_{60}$**: spectral changes following 1000 s of excitation (λ_{ex} = 480 nm, 0.8 mW) in CH_2Cl_2 (black: prior to excitation, red: after excitation).

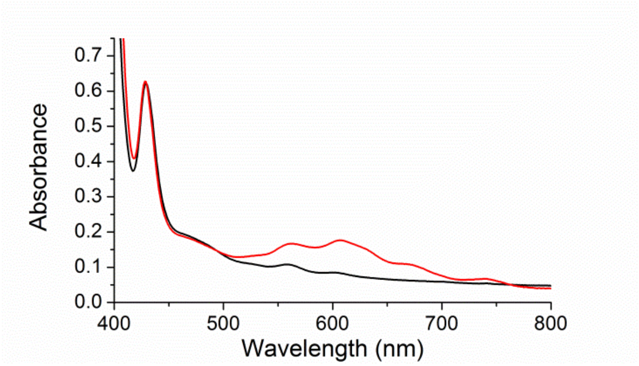

Supplementary Figure 35. Catalytic reduction of methyl viologen in the presence of 1 µM **1** and 10 eq. of **ImC$_{60}$**: spectral changes following 1000 s of excitation (λ_{ex} = 480 nm, 0.8 mW) in CH_2Cl_2 (black: prior to excitation, red: after excitation).

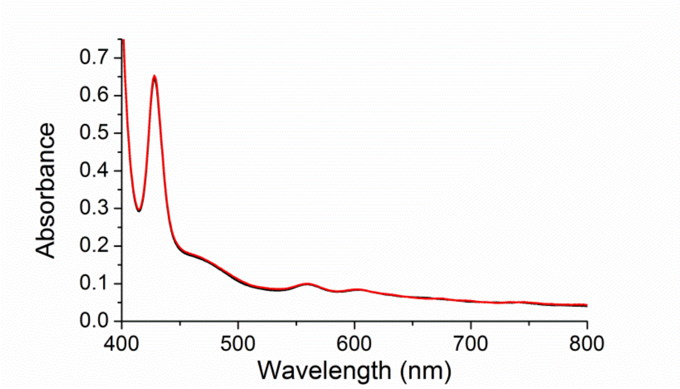

Supplementary Figure 36. Catalytic reduction of methyl viologen in the presence of 1 μM **3** and 10 eq. of **ImC$_{60}$**: spectral changes following 1000 s of excitation (λ_{ex} = 480 nm, 0.8 mW) in CH_2Cl_2 (black: prior to excitation, red: after excitation).

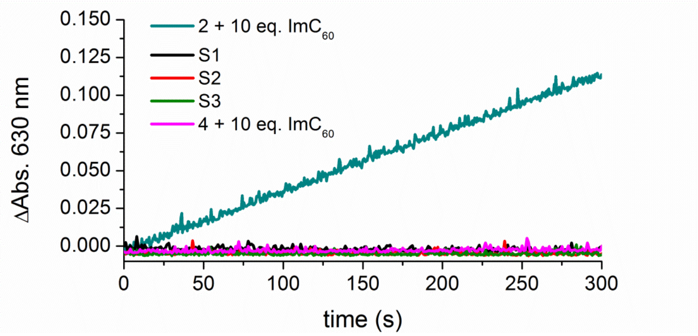

Supplementary Figure 37. Catalytic reduction of methyl viologen in the presence of 1 μM **1** and 10 eq. of **ImC$_{60}$** and control experiments with complexes **S1**, **S2**, **S3** and ligand **4** in the presence of 10 eq. of **ImC$_{60}$**: changes in absorbance at 630 nm (λ_{ex} = 480 nm, 0.8 mW) in CH_2Cl_2.

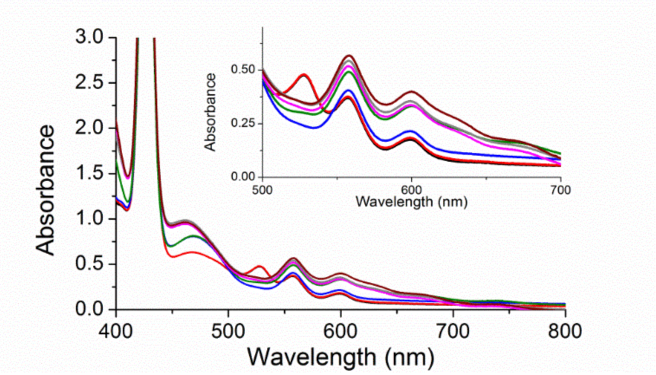

Supplementary Figure 38. Allosteric regulation of the reduction of methyl viologen in the presence of 5 μM **1** and 10 eq. of **ImC$_{60}$** (λ_{ex} = 480 nm, 0.2 mW, CH_2Cl_2): before excitation (black), 65 s excitation (red), addition of 1 drop of acetonitrile (blue), 165 s excitation (green), addition of 2 eq. of tetrabutylammonium chloride (pink), 65 s excitation (green), addition of 6 eq. of thalium triflate (grey), 165 s excitation (brown).

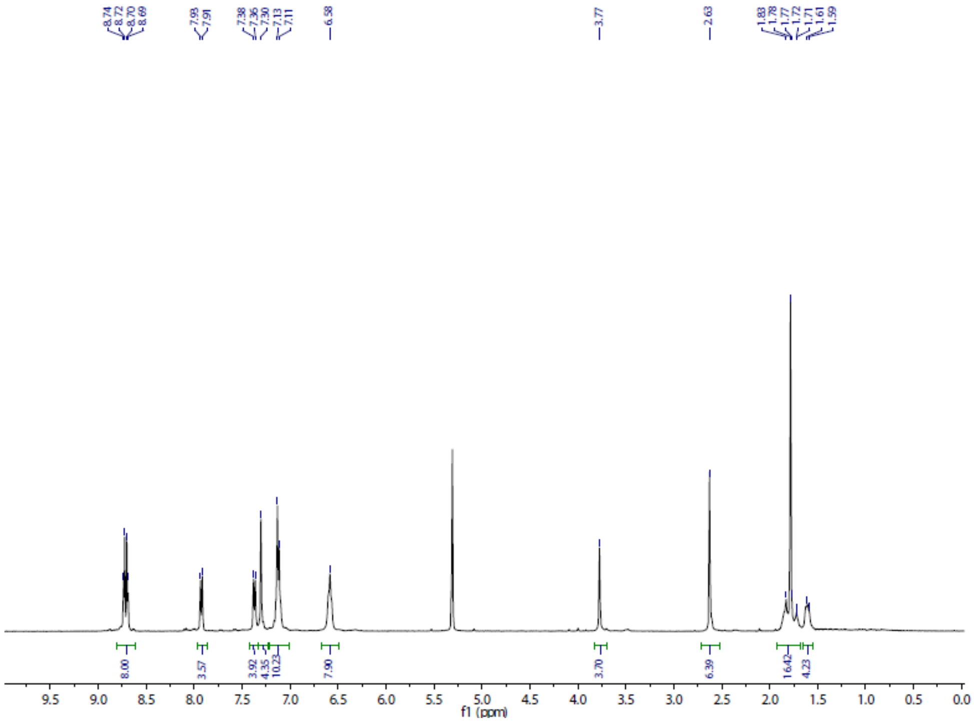

Supplementary Figure 39. ^{1}H NMR spectrum of **4** in CD_2Cl_2.

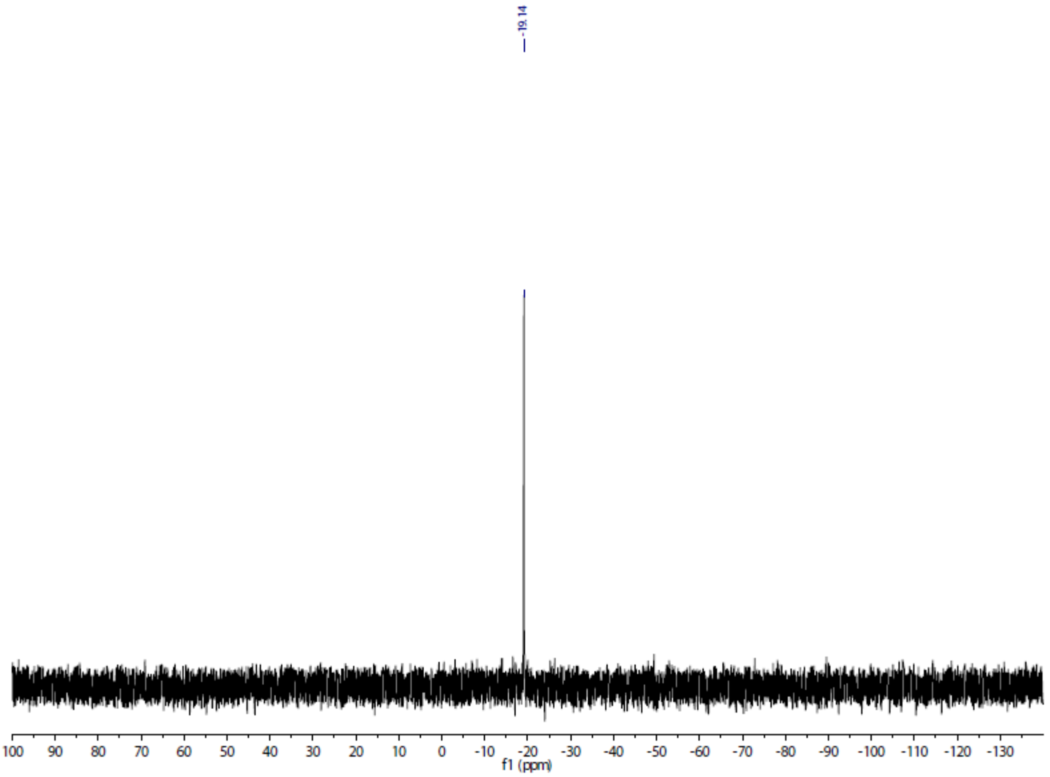

Supplementary Figure 40. $^{31}P\{^{1}H\}$ NMR spectrum of **4** in CD_2Cl_2.

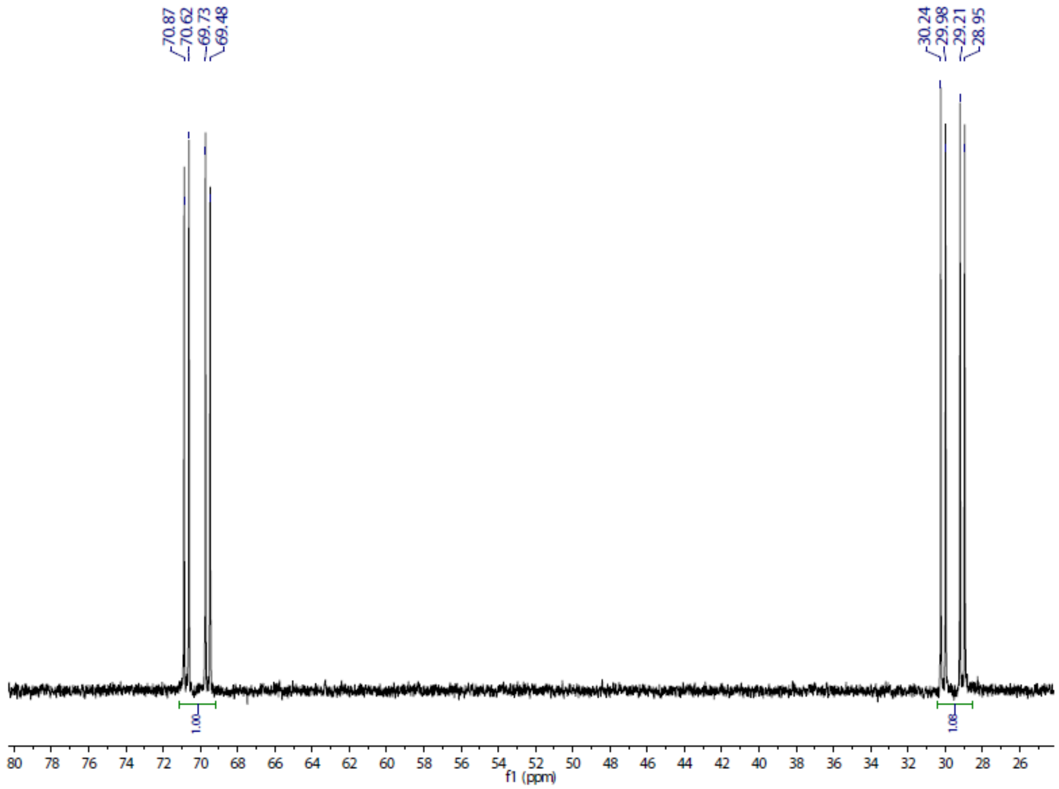

Supplementary Figure 41. $^{31}P\{^{1}H\}$ NMR spectrum of **S3** in CD_2Cl_2.

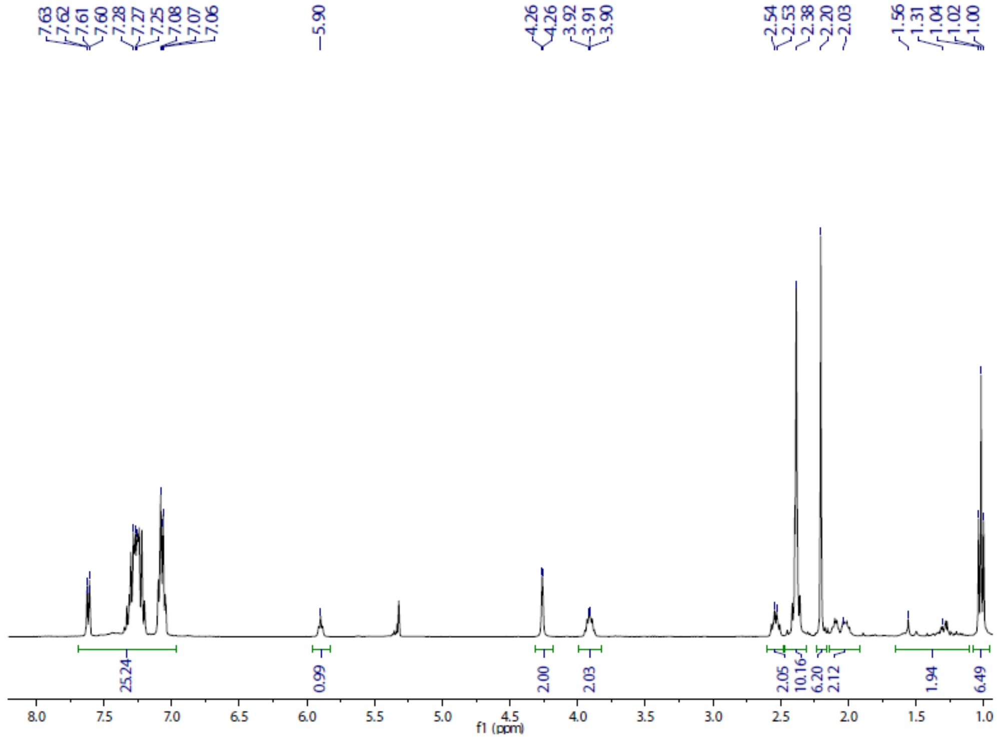

Supplementary Figure 42. ^{1}H NMR spectrum of **S3** in CD_2Cl_2.

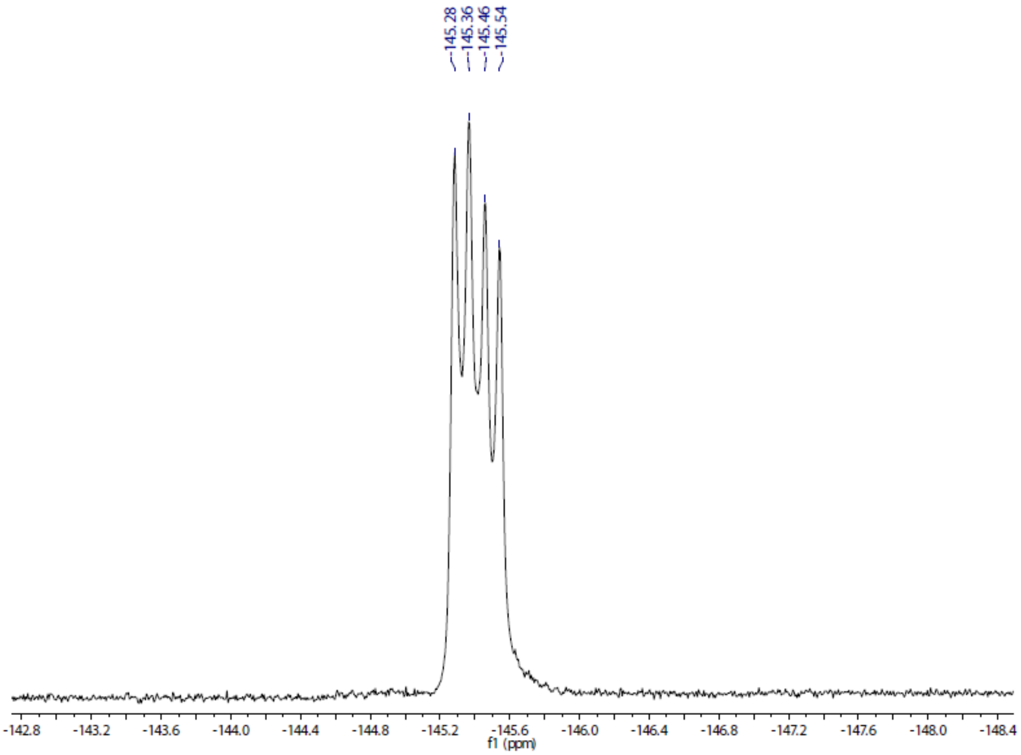

Supplementary Figure 43. ^{19}F NMR spectrum of **S3** in CD_2Cl_2.

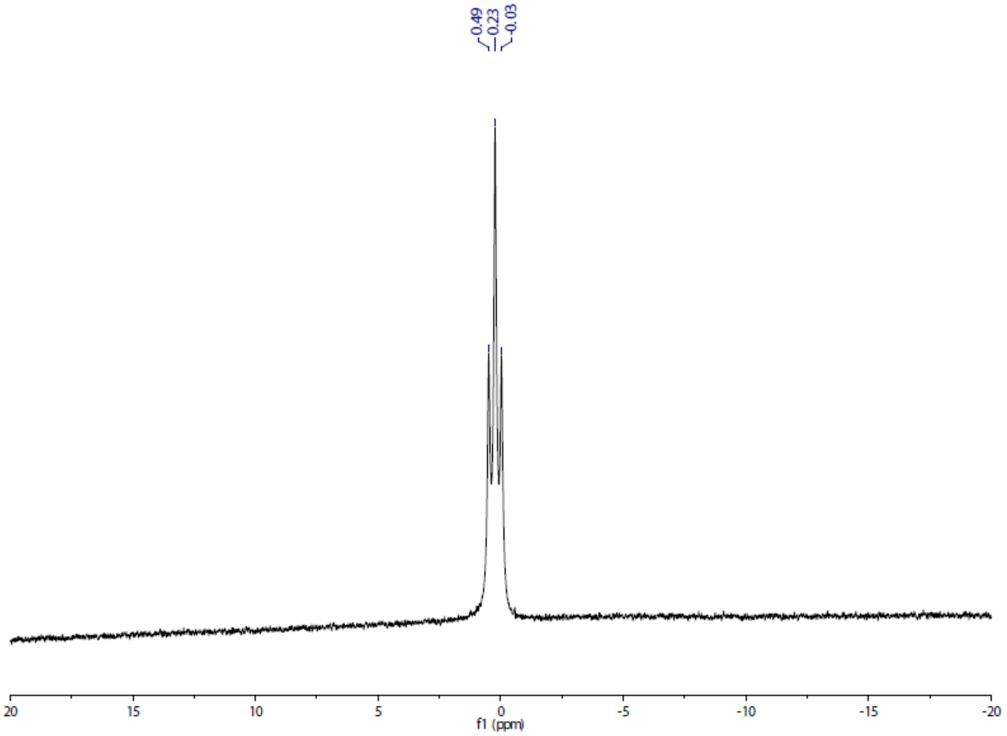

Supplementary Figure 44. $^{11}B\{^1H\}$ NMR spectrum of **S3** in CD_2Cl_2.

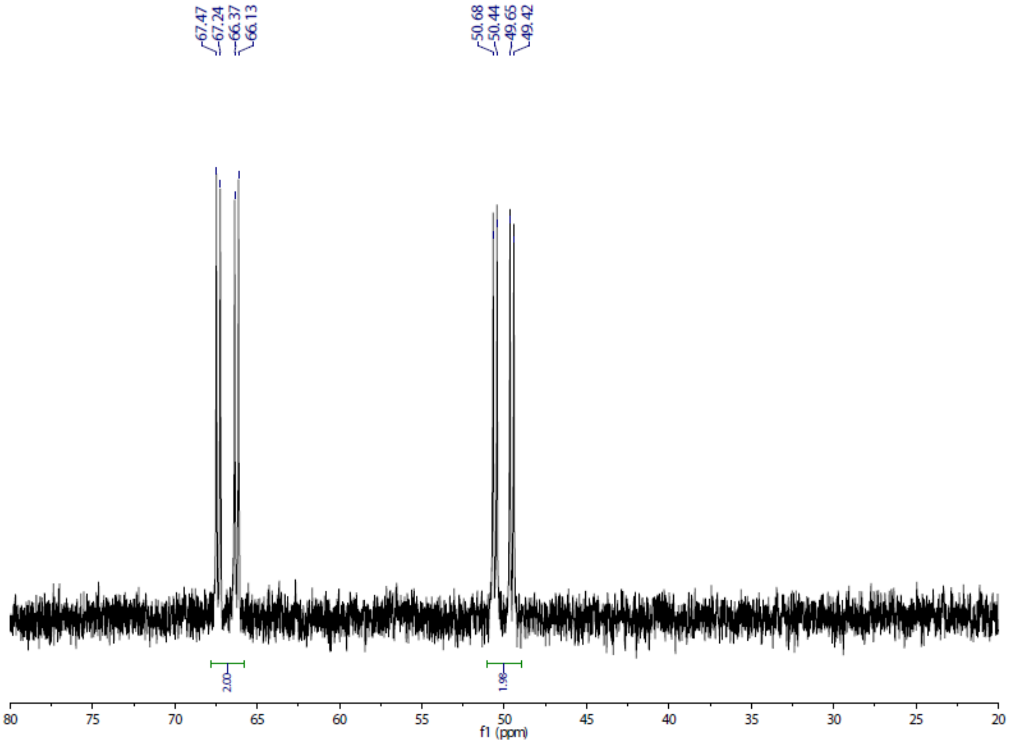

Supplementary Figure 45. $^{31}P\{^1H\}$ NMR spectrum of **1** in CD_2Cl_2 + 1 drop CD_3CN.

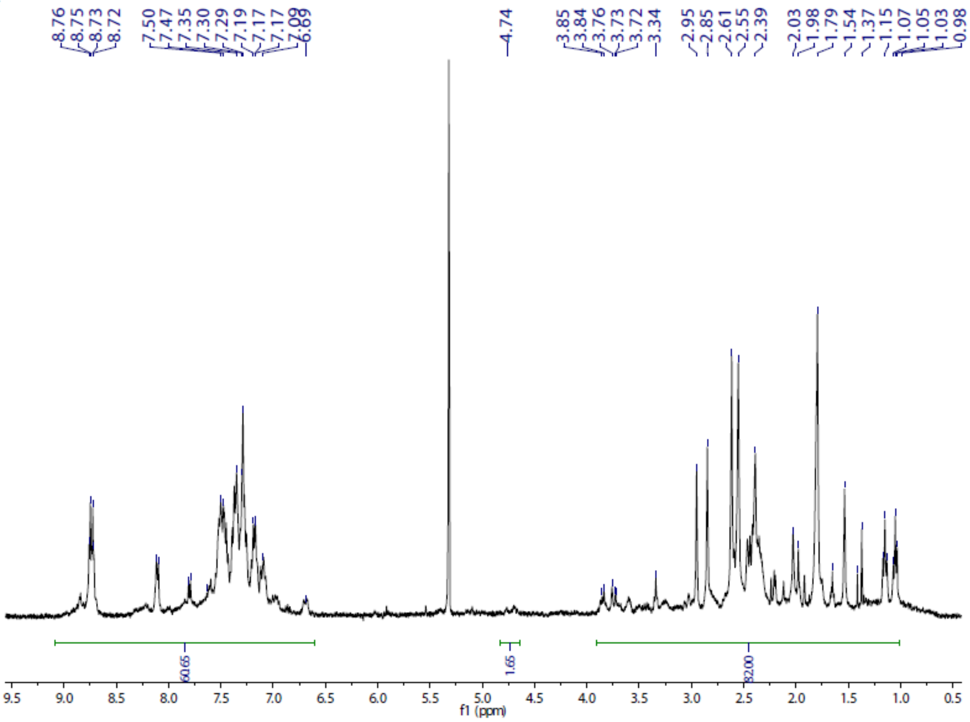

Supplementary Figure 46. ^1H NMR spectrum of **1** in CD_2Cl_2.

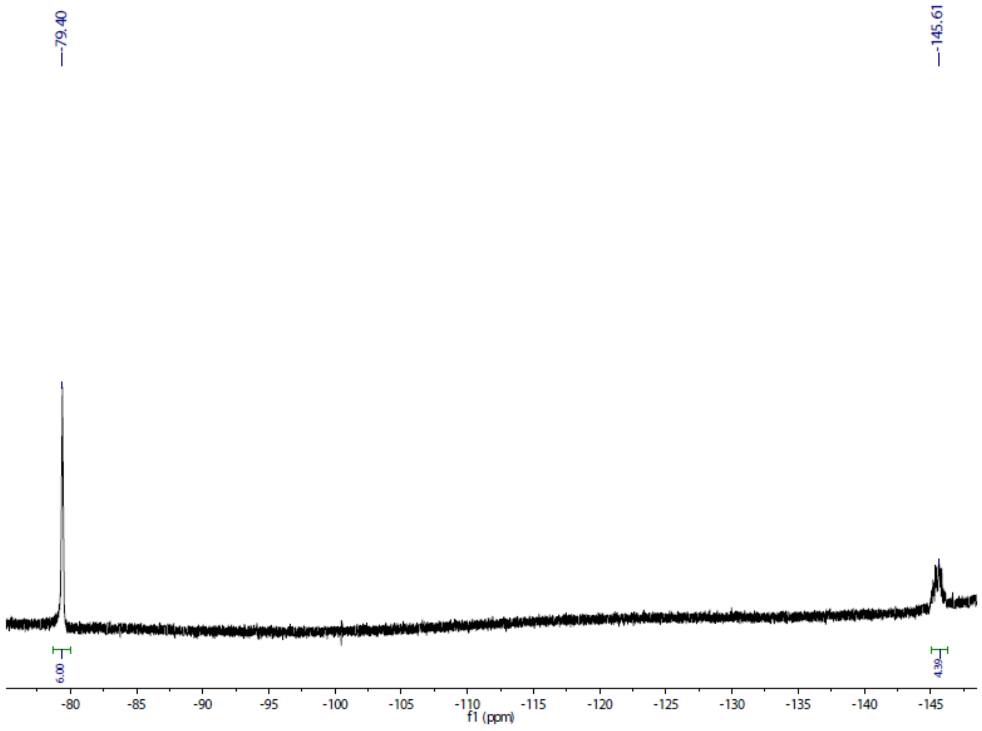

Supplementary Figure 47. ^{19}F NMR spectrum of **1** in CD_2Cl_2.

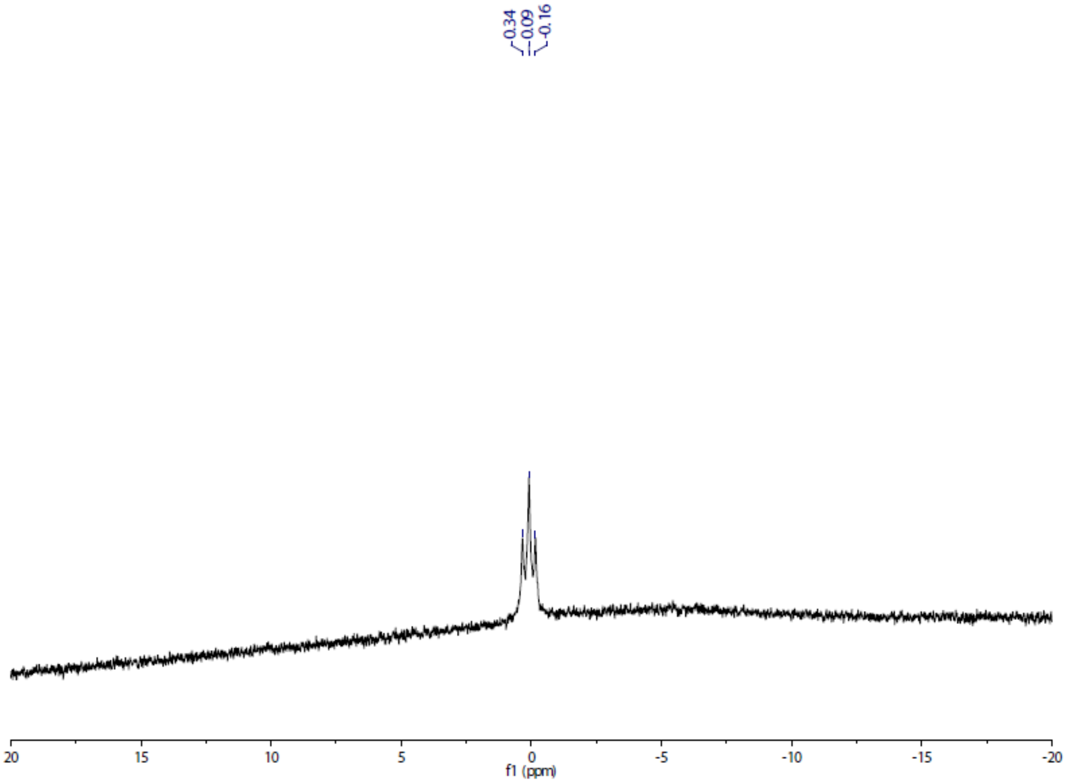

Supplementary Figure 48. $^{11}B\{^1H\}$ NMR spectrum of **1** in CD_2Cl_2.

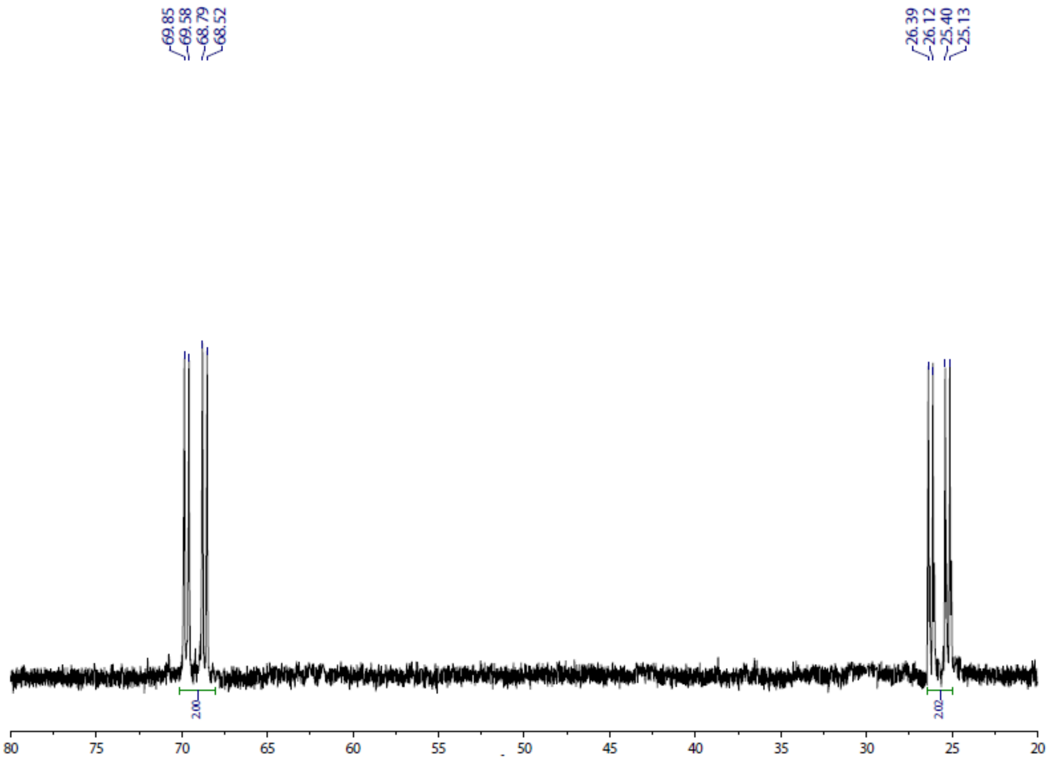

Supplementary Figure 49. $^{31}P\{^1H\}$ NMR spectrum of **2** in CD_2Cl_2 + 1 drop CD_3CN.

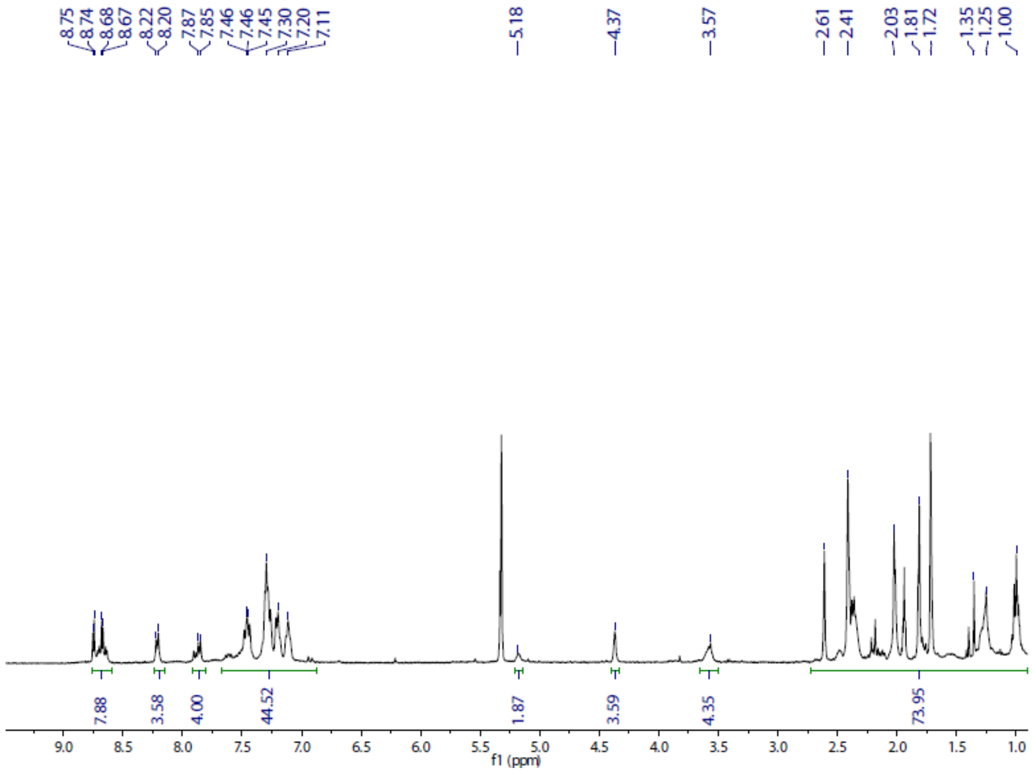

Supplementary Figure 50. ^1H NMR spectrum of **2** in CD_2Cl_2 + 1 drop CD_3CN.

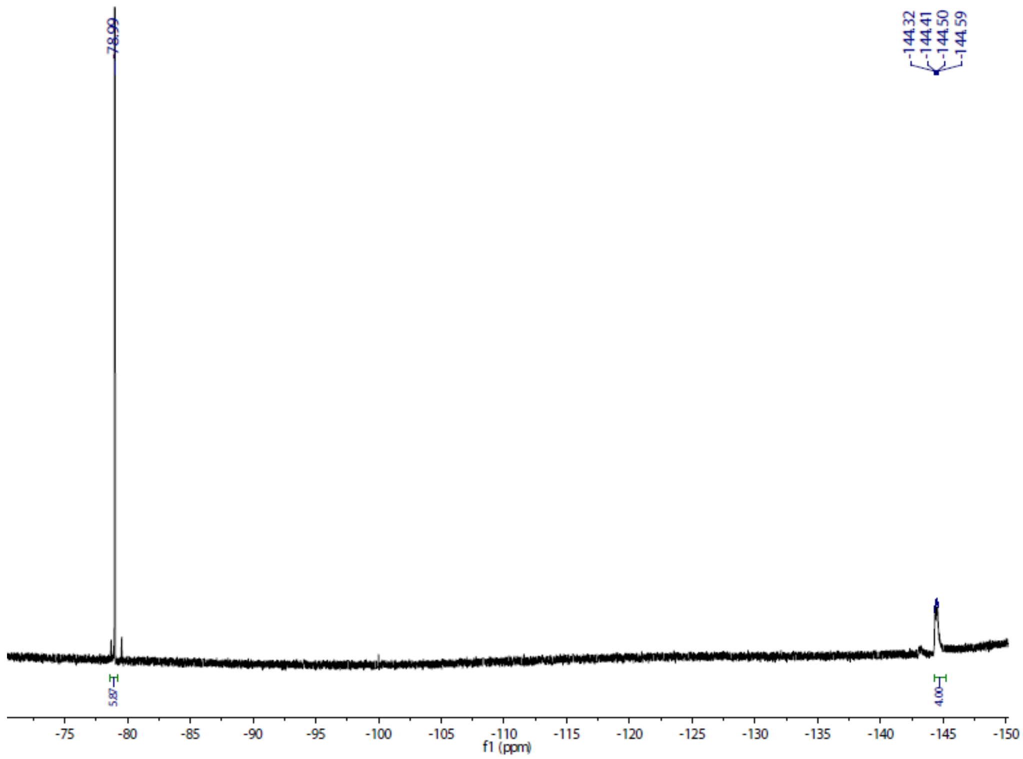

Supplementary Figure 51. ^{19}F NMR spectrum of **2** in CD_2Cl_2 + 1 drop CD_3CN.

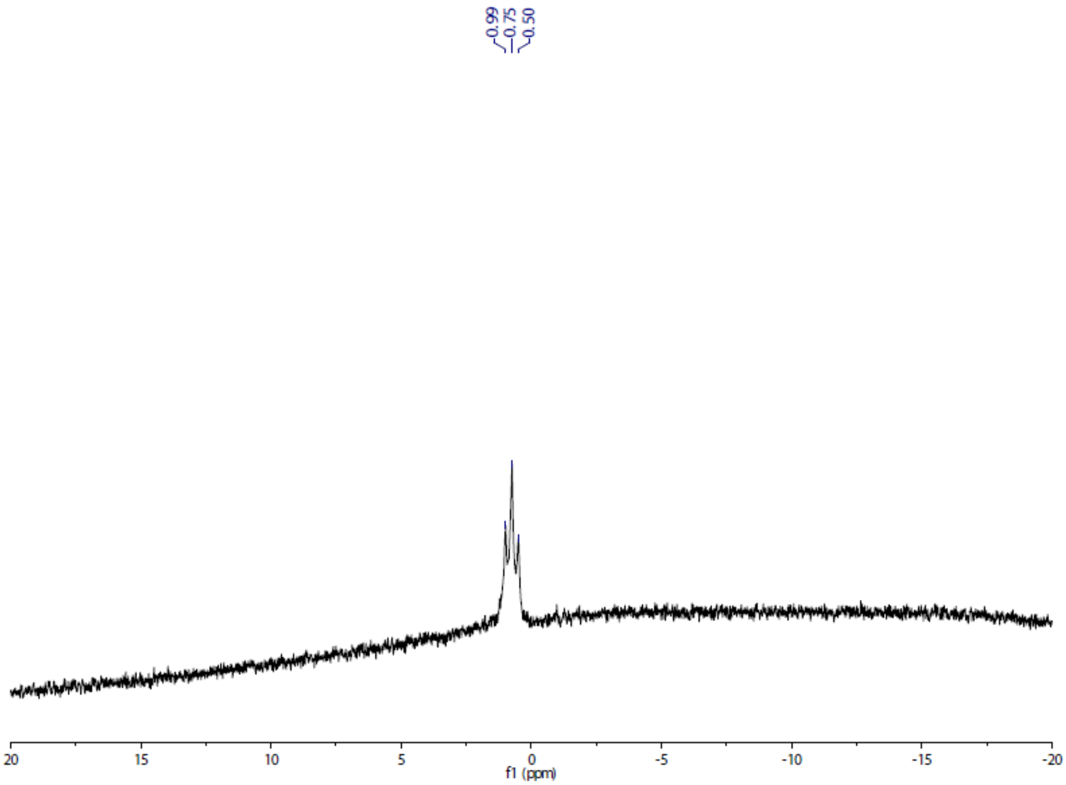

Supplementary Figure 52. $^{11}B\{^1H\}$ NMR spectrum of **2** in CD_2Cl_2 + 1 drop CD_3CN.

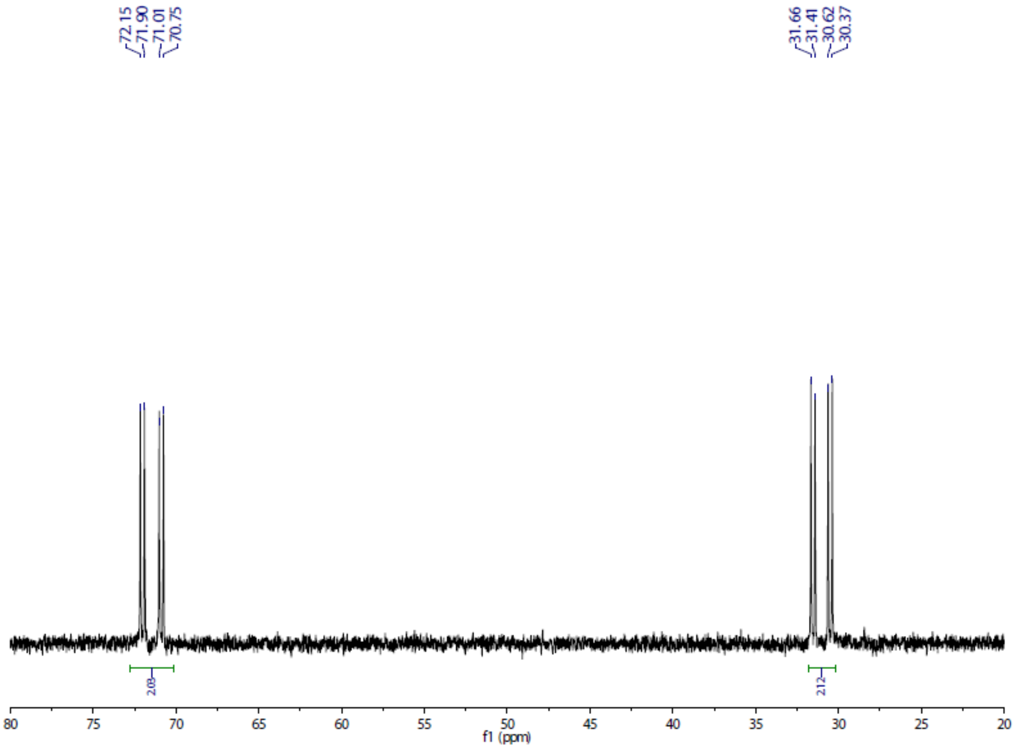

Supplementary Figure 53. $^{31}P\{^{1}H\}$ NMR spectrum of **3** in CD_2Cl_2.

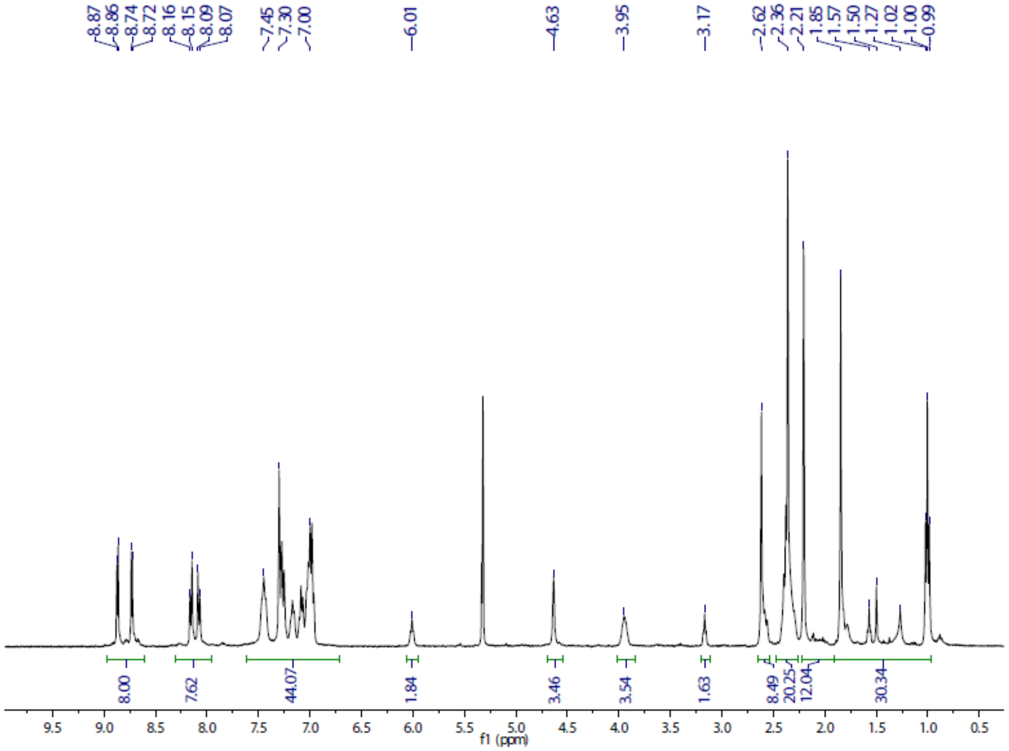

Supplementary Figure 54. ^{1}H NMR spectrum of **3** in CD_2Cl_2.

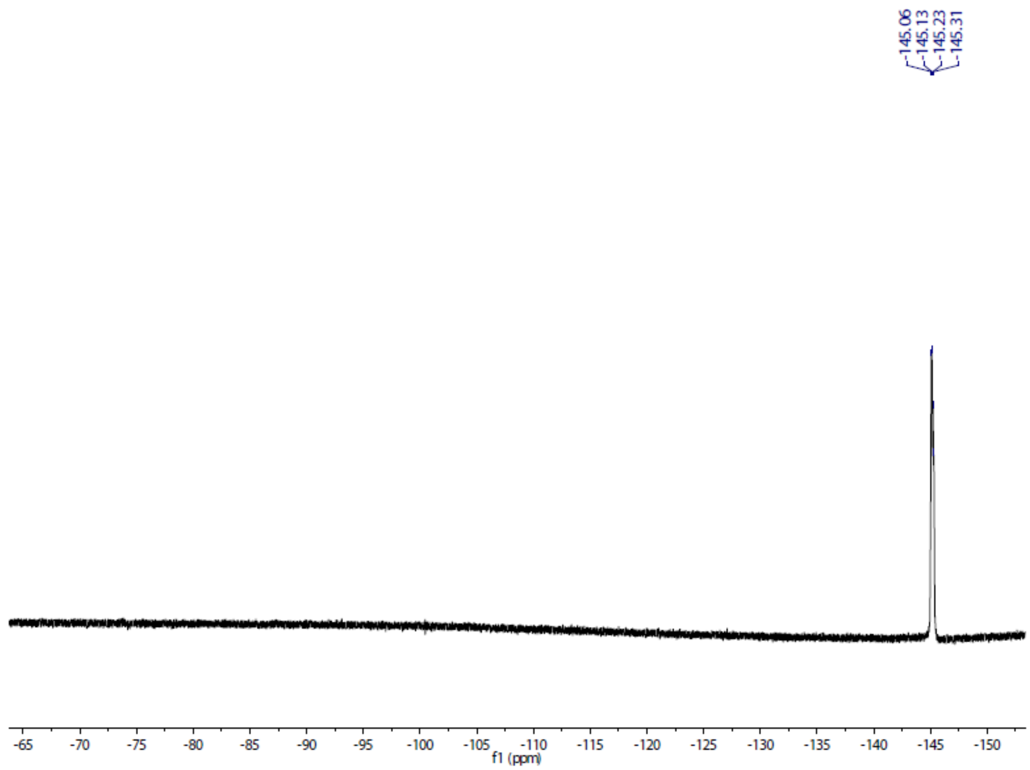

Supplementary Figure 55. ^{19}F NMR spectrum of **3** in CD_2Cl_2.

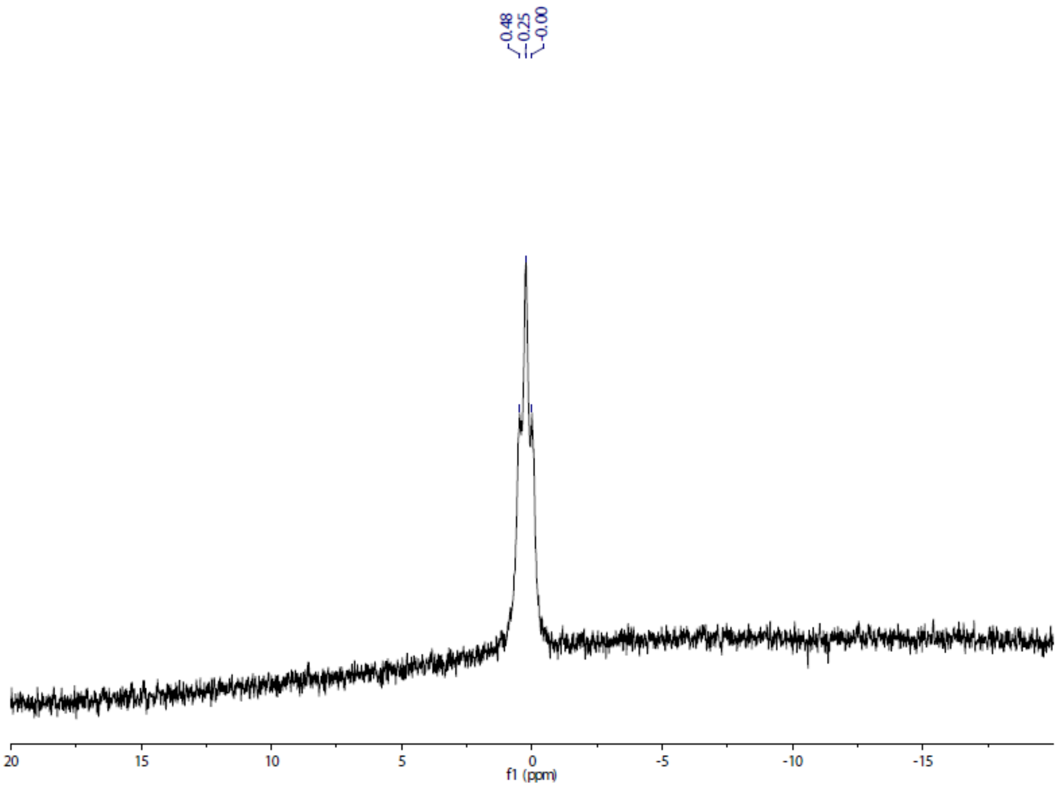

Supplementary Figure 56. $^{11}B\{^1H\}$ NMR spectrum of **3** in CD_2Cl_2.

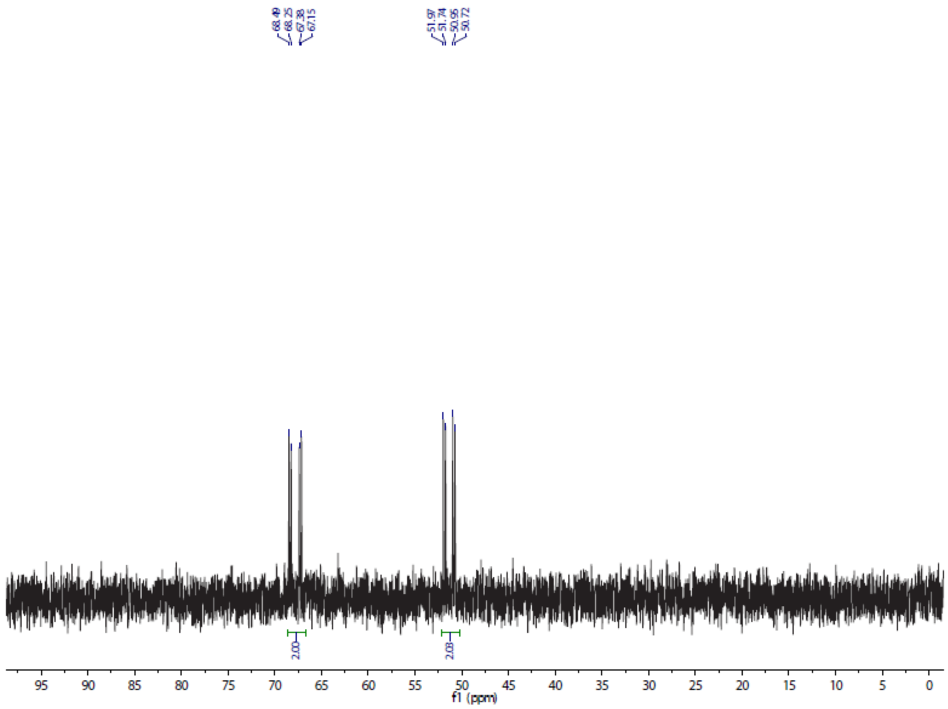

Supplementary Figure 57. $^{31}P\{^1H\}$ NMR spectrum of **1-ImC$_{60}$** in CD_2Cl_2.

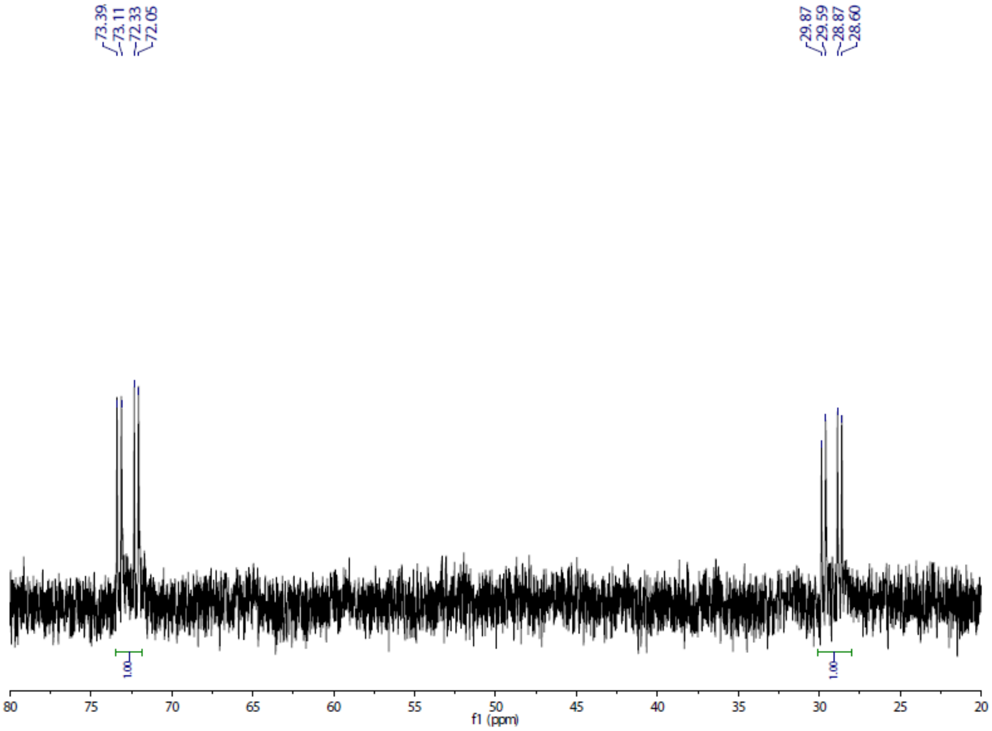

Supplementary Figure 58. $^{31}P\{^1H\}$ NMR spectrum of **2-ImC$_{60}$** in CD_2Cl_2 + 1 drop CD_3CN.

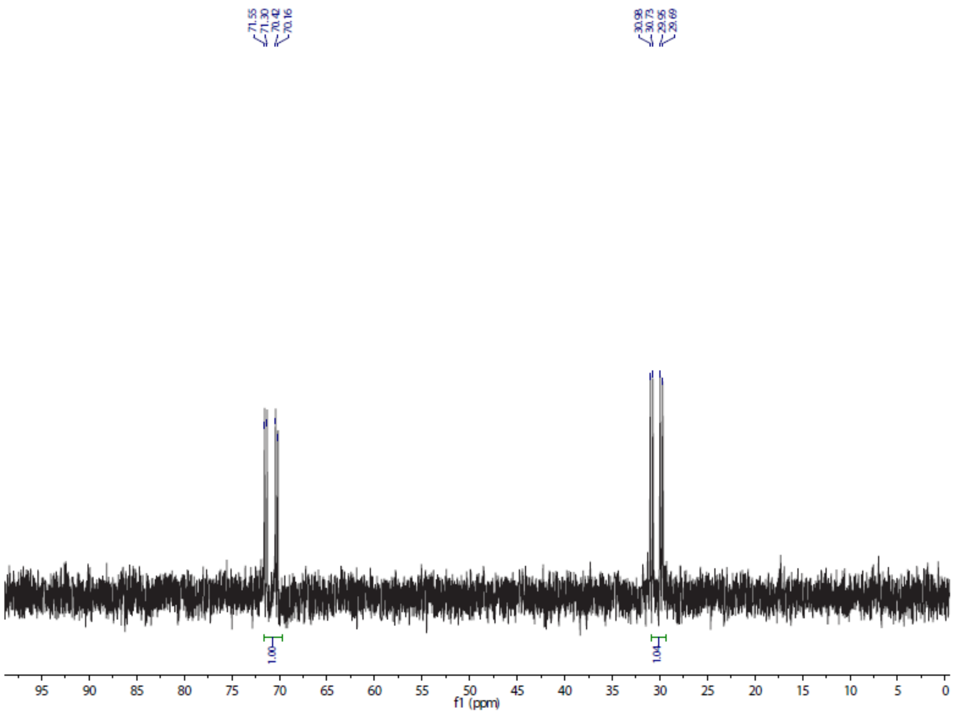

Supplementary Figure 59. $^{31}P\{^{1}H\}$ NMR spectrum of **3-ImC$_{60}$** in CD_2Cl_2.

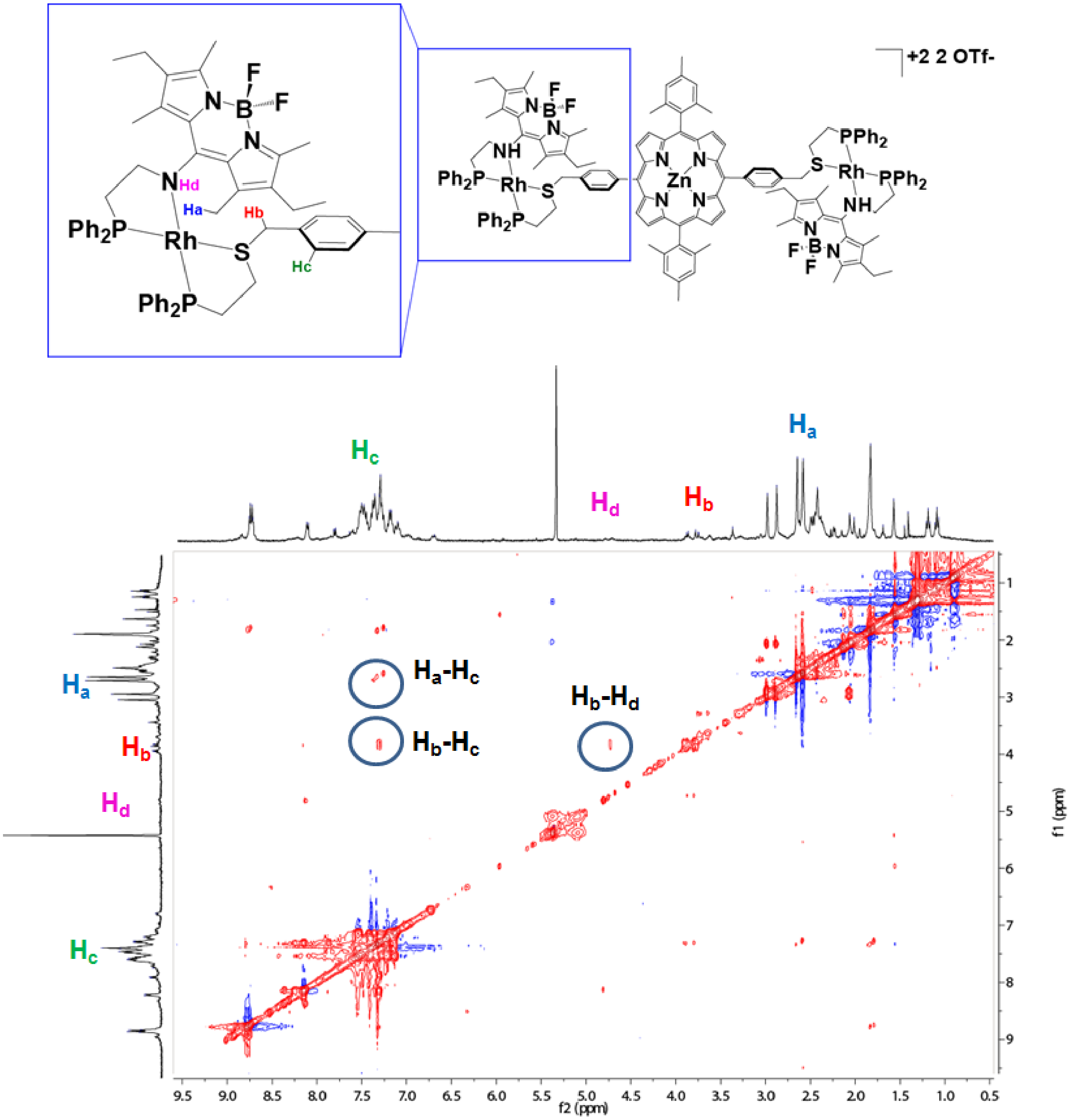

Supplementary Figure 60. ^{1}H NOESY NMR spectrum of **1** in CD_2Cl_2.

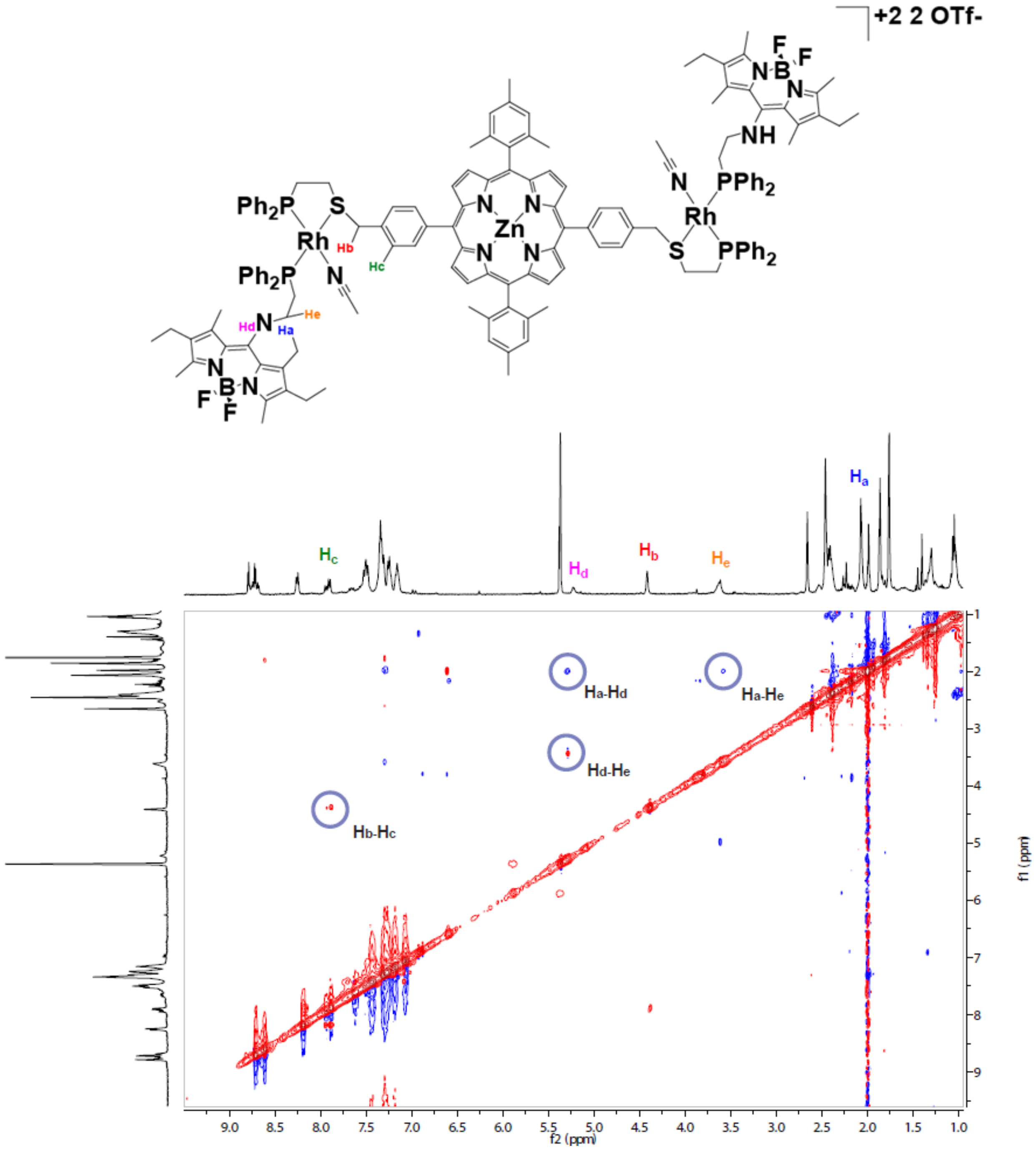

Supplementary Figure 61. ^{1}H NOESY NMR spectrum of **2** in CD_2Cl_2.

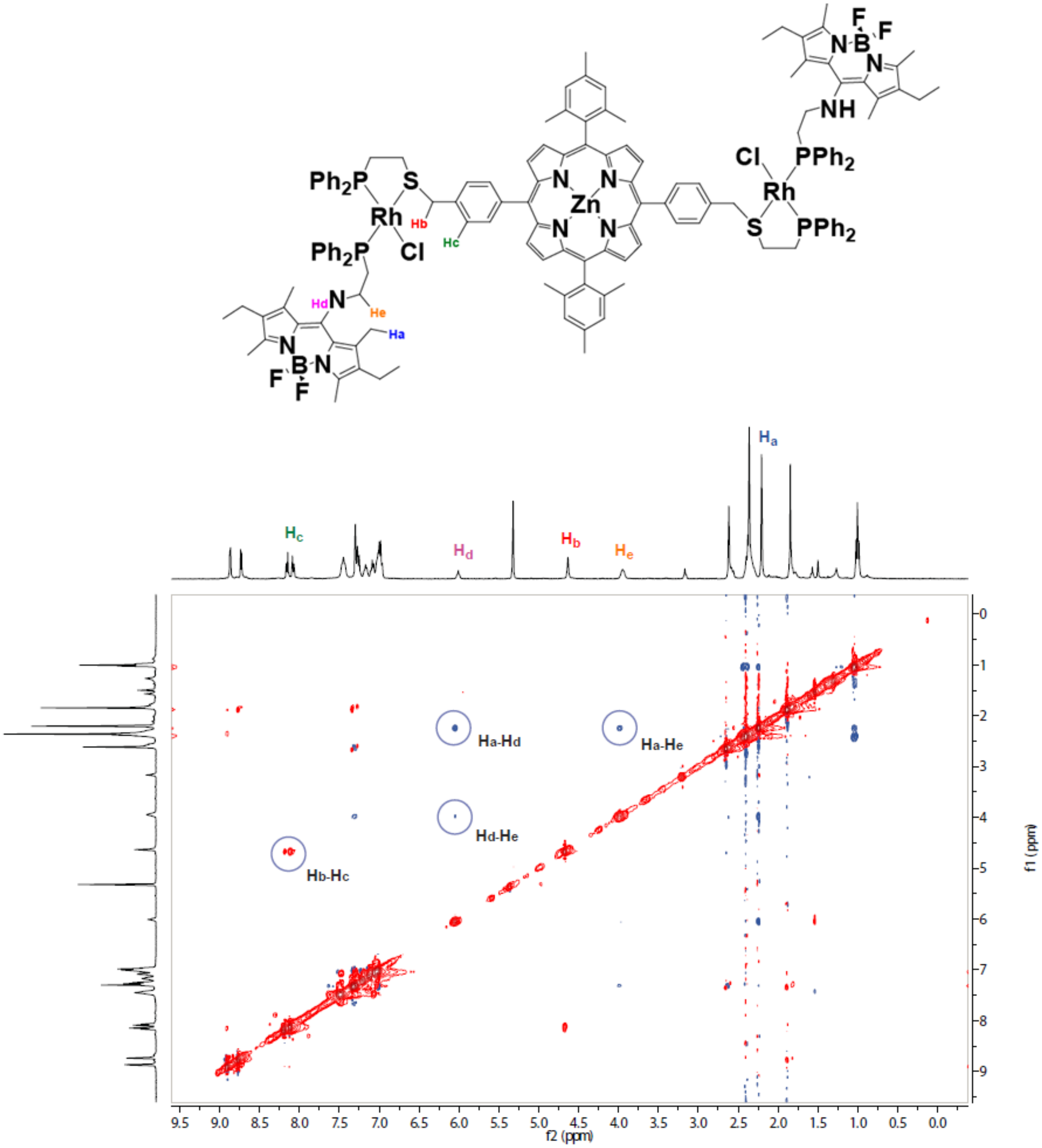

Supplementary Figure 62. ^{1}H NOESY NMR spectrum of **1** in CD_2Cl_2.

Supplementary Tables:

Supplementary Table 1. Electrochemical Data.

Complex	$E_{ox.\ an.}$ Bodipy (V)	$E_{ox.\ an.}$ Rh(I) (V)
**1**	0.186	0.062
**2**	0.005	0.098
**3**	0.032	-0.455

Supplementary Table 2. Optical and Kinetic Data.[a]

Complex	$\lambda_{max\ Bodipy}$ (nm)	$\lambda_{fluo.\ em.\ Bodipy}$ (nm)	$\tau_{1/2\ Bodipy}$ (ps)	$\tau_{1/2\ Porphyrin}$ (ps)	Q.Y.$_{total\ fluor.\ em.}$
**1**	465	517	79±12	1274±207	0.038
**2**	463	521	48±4	1126±67	0.031
**3**	463	522	44±6	1121±90	0.020
**6**	465	519	91±9	-	0.047
**7**	463	520	350±19	-	0.344
**8**	463	522	45±3	-	0.018

[a] Excited State half-life values were calculated from the time constants generated from the kinetic fits.

Supplementary Table 3. Crystallographic information.

Compound	**4**	**5**	**X1**
Empirical formula	C80H70N4P2S2Zn	C31H37BF2N3P	C156H142B2F18N8O6P4Rh2S6Zn
Formula weight	1278.83	531.41	3175.82
Temperature / K	100.05	100.03	100
Crystal system	monoclinic	monoclinic	triclinic
Space group	C2/c	P21/c	P-1
a / Å, b / Å, c / Å	28.4395(15), 18.7648(11), 16.3621(9)	8.4508(4), 41.4961(16), 8.0700(3)	15.6349(9), 18.4356(12), 21.7530(15)
α/°, β/°, γ/°	90, 123.051(3), 90	90, 94.122(4), 90	107.517(4), 110.288(4), 96.416(4)
Volume / Å3	7318.9(7)	2822.6(2)	5442.2(6)
Z	4	4	1
ρcalc / mg mm-3	1.161	1.251	0.969
μ / mm-1	1.745	1.169	2.62
F(000)	2680	1128	1630
Crystal size / mm3	0.393 × 0.142 × 0.041	0.15 × 0.098 × 0.021	0.147 × 0.063 × 0.023
2Θ range for data collection	5.98 to 130.08°	4.258 to 127.406°	5.18 to 101.698°
Index ranges	-33 ≤ h ≤ 33, -22 ≤ k ≤ 21, -19 ≤ l ≤ 18	-9 ≤ h ≤ 9, -48 ≤ k ≤ 42, -9 ≤ l ≤ 8	-15 ≤ h ≤ 15, -18 ≤ k ≤ 15, -10 ≤ l ≤ 21
Reflections collected	56300	10525	14784
Independent reflections	6214[R(int) = 0.0398]	4343[R(int) = 0.0769]	10661[R(int) = 0.0533]
Data/restraints/parameters	6214/0/406	4343/0/353	10661/960/871
Goodness-of-fit on F2	1.087	1.096	1.333
Final R indexes [I>2σ (I)]	R1 = 0.0541, wR2 = 0.1604	R1 = 0.0838, wR2 = 0.2054	R1 = 0.1434, wR2 = 0.3731
Final R indexes [all data]	R1 = 0.0608, wR2 = 0.1661	R1 = 0.1133, wR2 = 0.2168	R1 = 0.1940, wR2 = 0.4064
Largest diff. peak/hole / e Å-3	0.829/-0.614	0.781/-0.398	2.863/-1.700

Supplementary Methods:

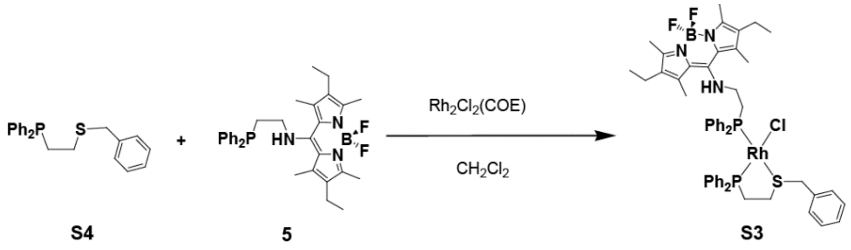

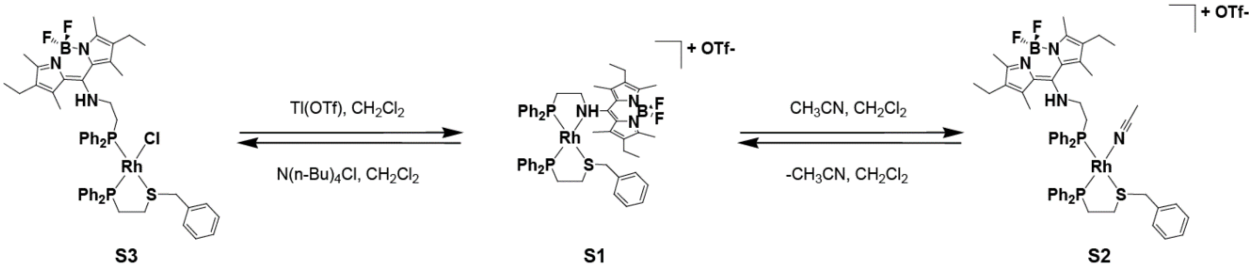

Synthesis of [RhCl(κ_2-P,S-Bz)(5)] (S3). A solution of P,S-Benzyl **S1** (33.6 mg, 0.100 mmol) in 5 mL of CH_2Cl_2 was added dropwise to a solution of Rh_2Cl_2(cyclooctene)$_4$ (35.9 mg, 0.100 mmol) in 5 mL of CH_2Cl_2. After stirring for 5 minutes, a solution of P.N-Bodipy ligand **5** (53.1 mg, 0.100 mmol) in 5 mL of CH_2Cl_2 was added in a dropwise fashion and the mixture was left to stir for 24 hours. The solution volume was then reduced to approximately 1 mL and the product was precipitated with pentane. The product was collected *via* vacuum filtration and washed with pentane to afford the semiopen complex (*in situ* $^{31}P\{^1H\}$ NMR yields = 80-85%, isolated yields 72.8 mg, 72%). ^1H NMR (400.16 MHz, 25˚C, CD_2Cl_2): δ 7.61 (d, J_{H-H} = 4 Hz, 2 H), 7.45-7.15 (m, 15 H), 7.12-7.00 (m, 8 H), 5.90 (m, 1 H), 4.26 (s, 2 H), 3.91 (m, 2H), 2.53 (m, 2H), 2.38 (m, 10 H), 2.20 (s, 6 H), 2.12-1.95 (m, 4 H), 1.02 (t, J_{H-H} = 8 Hz, 6 H). $^{31}P\{^1H\}$ NMR (161.98 MHz, 25˚C, CD_2Cl_2): δ 70.1 (dd, J_{P-P} = 41 Hz, J_{P-Rh} = 186 Hz, 1 P), 29.6 (dd, J_{P-P} = 42 Hz, J_{P-Rh} = 167 Hz, 1 P). ^{19}F NMR (376.49 MHz, 25˚C, CD_2Cl_2): δ -145.4 (q, J_{F-B} = 34 Hz, 2 F). $^{11}B\{^1H\}$ NMR (128.38 MHz, 25˚C, CD_2Cl_2): δ 0.23 (t, J_{B-F} = 33 Hz). HRMS (ESI+) *m/z* calcd for $[M]^+$: 1005.2631; found: 1005.2645.

Supplementary Reference:

1 Lifschitz, A. M. *et al.* Chemically regulating Rh(I)-Bodipy photoredox switches. *Chem. Commun.*, **50**, 6850-6852 (2014).
